# LYAR potentiates rRNA synthesis by recruiting BRD2/4 and the MYST-type acetyltransferase KAT7 to rDNA

**DOI:** 10.1093/nar/gkz747

**Published:** 2019-09-02

**Authors:** Keiichi Izumikawa, Hideaki Ishikawa, Harunori Yoshikawa, Sally Fujiyama, Akira Watanabe, Hiroyuki Aburatani, Hiroyuki Tachikawa, Toshiya Hayano, Yutaka Miura, Toshiaki Isobe, Richard J Simpson, Li Li, Jinrong Min, Nobuhiro Takahashi

**Affiliations:** 1 Department of Applied Life Science, Tokyo University of Agriculture and Technology, 3-5-8 Saiwai-cho, Fuchu-shi, Tokyo 183-8509, Japan; 2 Global Innovation Research Organizations, Tokyo University of Agriculture and Technology, 3-5-8 Saiwai-cho, Fuchu, Tokyo 183-8509, Japan; 3 Centre for Gene Regulation & Expression, School of Life Sciences, University of Dundee, Dow Street, Dundee DD1 5EH, UK; 4 Department of Life Science Frontiers, Center for iPS Cell Research and Application, Kyoto University 53, Shogoin-kawahara-cho, Sakyo-ku, Kyoto-shi, Kyoto 606-8507, Japan; 5 Laboratory for System Biology and Medicine, University of Tokyo, 4-6-1 Komaba, Meguro-ku, Tokyo 153-8904, Japan; 6 Department of Applied Life Science, The University of Tokyo, 1-1-1 Yayoi, Bunkyo-ku, Tokyo 113-8657, Japan; 7 Department of Biomedical Sciences, College of Life Sciences, Ritsumeikan University, 1-1-1 Nojihigashi, Kusatsu 525-8577, Japan; 8 Department of Chemistry, Graduate School of Sciences and Engineering, Tokyo Metropolitan University, 1-1 Minamiosawa, Hachiouji-shi, Tokyo 192-0397, Japan; 9 La Trobe Institute for Molecular Science (LIMS) LIMS Building 1, Room 412 La Trobe University, Bundoora Victoria 3086, Australia; 10 Structural Genomics Consortium, University of Toronto, 101 College St., Toronto, Ontario M5G 1L7, Canada; 11 Department of Physiology, University of Toronto, Toronto, Ontario M5S 1A8, Canada

## Abstract

Activation of ribosomal RNA (rRNA) synthesis is pivotal during cell growth and proliferation, but its aberrant upregulation may promote tumorigenesis. Here, we demonstrate that the candidate oncoprotein, LYAR, enhances ribosomal DNA (rDNA) transcription. Our data reveal that LYAR binds the histone-associated protein BRD2 without involvement of acetyl-lysine–binding bromodomains and recruits BRD2 to the rDNA promoter and transcribed regions *via* association with upstream binding factor. We show that BRD2 is required for the recruitment of the MYST-type acetyltransferase KAT7 to rDNA loci, resulting in enhanced local acetylation of histone H4. In addition, LYAR binds a complex of BRD4 and KAT7, which is then recruited to rDNA independently of the BRD2-KAT7 complex to accelerate the local acetylation of both H4 and H3. BRD2 also helps recruit BRD4 to rDNA. By contrast, LYAR has no effect on rDNA methylation or the binding of RNA polymerase I subunits to rDNA. These data suggest that LYAR promotes the association of the BRD2-KAT7 and BRD4-KAT7 complexes with transcription-competent rDNA loci but not to transcriptionally silent rDNA loci, thereby increasing rRNA synthesis by altering the local acetylation status of histone H3 and H4.

## INTRODUCTION

The ribosome is the essential cellular machinery for protein synthesis. The eukaryotic ribosome consists of a large 60S subunit and a small 40S subunit, the biogenesis of which requires transcription of a large ribosomal RNA precursor (47S pre-rRNA) by RNA polymerase (RNAP) I, a 5S rRNA by RNAP III, and translation of ribosomal proteins from mRNAs transcribed by RNAP II (1–6). In addition, numerous small nucleolar RNAs and non-ribosomal proteins (called *trans-*acting factors) participate in ribosome biogenesis (5,6). Among these factors, many contribute to the synthesis of 47S pre-rRNA, which alone constitutes ∼80% of the total transcription of proliferating cells (1,2). Because of its predominance, rDNA transcription is regulated by ≥30 *trans-*acting factors, including proto-oncogene products (MYC, etc.) and tumor suppressors (p53, retinoblastoma, etc.) to maintain appropriate ribosome numbers in normal cells (1–3,7). Upon oncogenic transformation of a cell, however, rRNA synthesis is upregulated by altered function of these types of factors, e.g. owing to their dysregulated expression and/or mutation (1). In certain tumor cells, the upregulation of rRNA synthesis occurs *via* phosphorylation of upstream binding factor (UBF), a rDNA promoter–binding protein that mediates binding of selectivity factor 1 (SL1) to the rDNA promoter, resulting in the recruitment of RNAP I. The increased activity of casein kinase II or of complexes formed between the mitotic cyclins and the cyclin-dependent kinases (CDK4-cyclin D1, CDK2-cyclin E) or the mitogen-activated protein kinase ERK (extracellular signal–regulated kinase) is responsible for this upregulation in certain cell types (1,2,7). For instance, casein kinase II phosphorylates the serine-rich carboxyl-terminal acidic tail of UBF, thereby promoting its interaction with SL1 (8). Failure to interrupt the binding between SL1 and UBF is another mechanism underlying the upregulation of rDNA transcription in cancer cells, which is often caused by inactivating mutations of the tumor-suppressor protein retinoblastoma or p53 (7,8). Another mechanism of rDNA upregulation occurs through increased expression of MYC along with the sequence-specific DNA-binding protein Max, which associates with CACGTG in the rDNA promoter and activates RNAP I–dependent transcription *via* recruitment of transformation/transcription domain–associated protein that bridges between MYC and the histone acetyltransferase GCN5, resulting in local acetylation of nucleosomal histones in rDNA loci (9–11). In addition, rRNA synthesis is accelerated at the level of transcriptional elongation by the actions of chromatin-remodeling and elongation complexes including the FACT and PAF complexes (12,13) and/or histone chaperones including nucleolin (14).

LYAR, the human ortholog of the mouse nucleolar protein LYAR (Ly-1 antibody-reactive clone), is a 45-kDa protein with 379 amino acid residues including a zinc-finger motif and three nuclear localization signals (15). LYAR is conserved across many species, including mammals. Mouse *LYAR* mRNA is detectable in immature spermatocytes and testes in early embryos and to a lesser extent in fetal liver and thymus tissue (15–17). In adult mice, the mRNA is detectable at low levels in kidney and spleen but not in other differentiated tissues including brain; however, the mRNA is expressed at very high levels in a number of mouse B- and T-cell leukemia lines (15). Mouse fibroblasts overexpressing mouse *LYAR* cDNA contribute to tumor formation in nude mice; thus, LYAR is believed to be a nucleolar oncoprotein that regulates the growth and proliferation of cancer cells (15). In support of this idea, LYAR is overexpressed in human medulloblastoma, the most common brain tumor in children (18), and it is also highly expressed in human metastatic colorectal cancer cells (19). Moreover, when overexpressed *in vitro*, LYAR increases the already potent proliferative capacity of HeLa cells (human uterine cervical cancer) and MCF cells (human breast cancer) (20).

Moreover, mouse LYAR is necessary for the expression of the fetal globin gene *via* its ability to associate with protein arginine methyl transferase 5 (21), and it is indispensable for cell proliferation and development of female mouse embryos in which *TP53* (encoding p53) is experimentally disrupted (16,22). Mouse LYAR also associates with nucleolin, a trans-acting factor involved in various stages of ribosome biogenesis, and together these proteins help maintain the pluripotency of embryonic stem cells (23). In addition, LYAR can bind to immature ribosome particles as well as a number of nucleolar proteins, including nucleophosmin (also known as B23), DDX21, UBF, and treacle, which is the product of *TCOF1*, the causative gene for Treacher Collins syndrome (24–32). LYAR also participates in pre-rRNA processing (20). Although modulation of rRNA synthesis is linked directly to differentiation of various stem-cell types including human embryonic stem cells (33–35), the involvement of LYAR in rRNA synthesis remains to be determined.

To address this shortcoming, we investigated LYAR function in human cells and found that LYAR binds rDNA *via* UBF and enhances pre-rRNA synthesis *via* its ability to mobilize the histone-associated proteins, the BRDs, to rDNA. UBF binds to the active nucleolar organizer region of genes, interacts directly with RNAP I (36), and has many functions during rDNA transcription, including the pause and release of RNAP I from the promoter (37). BRD2 enhances the recruitment of the acetyltransferase KAT7 and BRD4 to rDNA. Both BRD2 and BRD4 belong to the BET family of proteins and contain two tandem bromodomains and an extra-terminal domain (38). They are involved in RNAP II–dependent transcription and are recruited to euchromatin regions *via* their interaction with acetylated histones H3 and H4 (38,39). BRD2, which was originally identified as a Ser/Thr kinase (40,41), is necessary for the acetylation of lysine (K) residues of H4 (42), and BRD2 has histone chaperone activity that is required for RNAP II–dependent transcription of certain genes including *cyclin A* (42). BRD4 is a global regulator of p-TEFb (positive transcription elongation factor b)-dependent phosphorylation of RNAP II (43) and was recently found to be a histone acetyltransferase specific for histones H3 and H4, including H3K122 (44). Despite the roles of BRD family proteins in RNAP II–dependent transcription, their involvement in RNAP I–dependent transcription is unknown. On the other hand, KAT7, a member of a family of the MYST-type histone acetyltransferases, is required for acetylation of histones H3 and H4 depending on which co-binding partner is involved (JADE1, etc.) and for expression of development-specific genes in mammalian embryos (45). Our results suggest a mechanism by which RNAP I–dependent rRNA synthesis is regulated by LYAR, BRD proteins, and KAT7 in human cells.

## MATERIALS AND METHODS

### Metabolic labeling of newly synthesized 47S/45S pre-rRNA

The incorporation of 4-thiouridine and subsequent biotinylation were performed as described (46) with some modifications, noted as follows. HeLa or 293T cells in 35-mm Petri dishes were cultured in the presence of 10 μM 4-thiouridine (Sigma) for 30 min. Cells were collected with ice-cold PBS, and total RNA was isolated with TRIzol Reagent (Invitrogen). For the biotinylation of 4-thiouridine–labeled RNA, 20 μg of total RNA was incubated for 3 h at 25°C with 0.2 mg/ml biotin-coupled N-[6-(biotinamide)hexyl]-3’-(2’-pyridyldithio)propionamide (Pierce, 1 mg/ml in dimethylformamide) in 200 μl of 10 mM Tris–HCl, pH 7.4, containing 1 mM EDTA. An equal volume of chloroform was added, mixed, and incubated for 5 min. The mixture was subjected to centrifugation at 20,000 × *g* for 5 min, and the biotinylated RNA was precipitated from solution with isopropanol. After washing with 75% ethanol, each biotinylated RNA sample was dried and dissolved in formamide. For the detection of biotinylated 47S/45S pre-rRNA, 4 μg total RNA was electrophoresed on a 0.9% agarose/formaldehyde gel in MOPS running buffer at 3.5 V/cm for 3 h. Separated RNAs were transferred to a Hybond N+ membrane, which was subsequently dried, UV crosslinked using the CX-2000 UV Crosslinker (UVP, CA, USA) at 120 mJ/cm^2^, soaked in 10% acetic acid, and stained with methylene blue. Biotinylated 47/45S pre-rRNA was detected with horseradish peroxidase (HRP)–conjugated streptavidin using the Chemiluminescent Nucleic Acid Detection Module (Thermo Scientific). Signals were detected with the LAS-4000 system. Signal intensities of RNA bands were measured using NIH ImageJ software. To ensure equal loading, control RNA in each gel was stained using SYBR GOLD (Invitrogen) and quantified after the detection with the LAS-4000 system.

All other materials and methods are provided in supplementary materials including the list of antibodies (Supplementary Table S1) and that of DNA primers and small interference RNAs (Supplementary Table S2) used in this study.

## RESULTS

### *LYAR* is overexpressed in many human cancer cells

LYAR is a candidate oncoprotein (15), so we assessed the intracellular levels of LYAR or *LYAR mRNA* in tissues from normal/control subjects and cancer patients. In many of the tumor samples from cancer patients, including those with colorectal carcinoma or small-cell lung carcinoma, *LYAR* mRNA levels were higher than in normal tissues (Supplementary Figure S1A). In tumor samples from patients with colorectal carcinoma, *LYAR* mRNA levels, as determined by GeneChip, were higher than those in normal cells from those patients as well as in cells from healthy subjects (Supplementary Figure S1B). In addition, we examined *LYAR* mRNA levels in the low-metastasis colorectal cancer cell line SW480 and the highly metastatic line SW620 in comparison with HeLa cells. These cell lines were established from the primary lesion (SW480) and lymph-node metastases (SW620) of a single colorectal cancer patient (47,48). SW620 cells expressed a much higher level of both LYAR protein and mRNA than did SW480 cells (Supplementary Figure S1C).

### LYAR associates with rDNA and increases the expression of 47S/45S pre-rRNA

**Figure 1.**
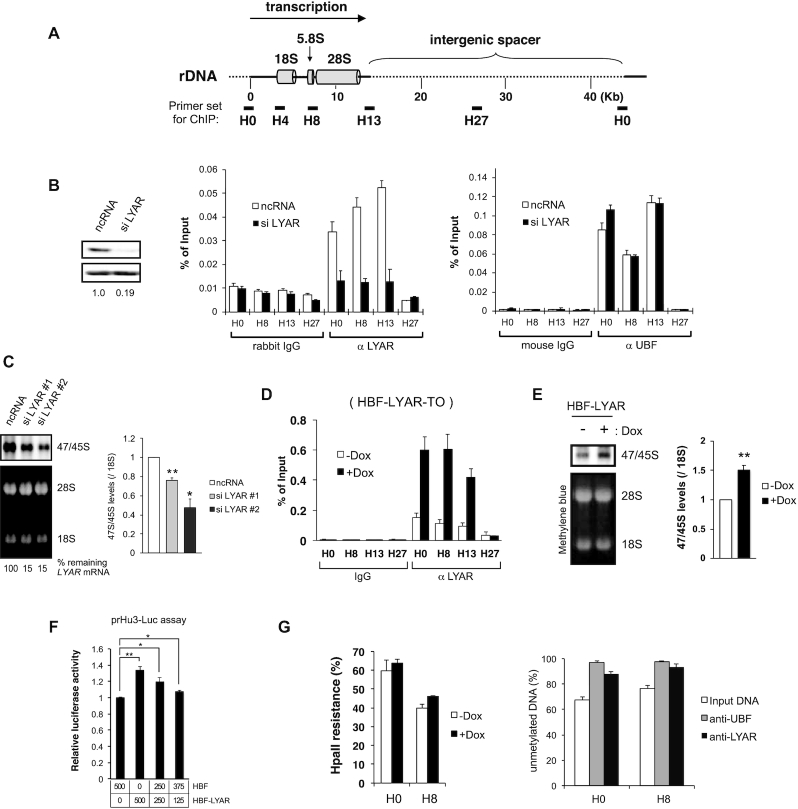
LYAR is involved in rDNA transcription. (**A**) Schematic diagram of a single human rDNA genic region (rDNA repeats are separated by an intergenic spacer). Primer sets (short bars: subregions H0, H8, H13, and H27) used for ChIP analysis are indicated along with their approximate positions relative to the transcription start site (0 kb). (**B**) ChIP analysis of UBF and LYAR binding to rDNA loci in HeLa cells transfected with scRNA (control) or siRNA specific for LYAR. Nonspecific mouse IgG or rabbit IgG was used as the antibody control. The chromatin-immunoprecipitated (ChIPed) DNA was quantified by qPCR using the primer sets indicated in A. The graph shows the amount of ChIPed DNA (% of input). Data represent the mean ± SEM of three independent experiments. The efficiency of LYAR knockdown was assessed with immunoblotting. (**C**) Metabolic labeling (4-thiouridine) of newly synthesized 47/45S pre-rRNA in HeLa cells upon LYAR knockdown. The RNA extracted for the cells treated with siRNA #1, siRNA #2 or ncRNA specific for LYAR (or ncRNA, control), was biotinylated and then subjected to agarose gel electrophoresis under denaturing condition and northern blotting. Signals for 47/45S pre-rRNA were detected by chemiluminescence. 28S and 18S rRNAs were used as loading controls (stained with methylene blue). The graph shows the relative band intensities of biotin-labeled 47/45S pre-rRNA normalized to that of 18S rRNA. Data reflect the mean ± SEM of three independent experiments. *P < 0.05 (paired t-test). *LYAR* mRNA levels, normalized to *GAPDH* mRNA, were assessed by reverse transcription-qPCR. (**D**) ChIP analysis of LYAR binding to rDNA loci (H0, H8, H13, H27) in HBF-LYAR-TO cells with or without HBF-LYAR induction *via* Dox. Nonspecific rabbit IgG was used as the antibody control. The graph shows the amount of ChIPed DNA (% of input). Data reflect the mean ± SEM of three independent experiments. (**E**) Metabolic labeling (4-thiouridine) of newly synthesized 47/45S pre-rRNA in HBF-LYAR-TO cells with or without Dox treatment. The pre-rRNA was biotinylated and then subjected to agarose gel electrophoresis under denaturing condition and northern blotting. Signals for 47/45S pre-rRNA were detected by chemiluminescence. 18S rRNAs were used as loading controls (stained with methylene blue). The graph shows the relative band intensities of 2 biotin-labeled 47/45S pre-rRNA normalized to that of 18S rRNA. Data reflect the mean ± SEM of six independent experiments. **P < 0.01 (paired t-test). (**F**) prHu3-Luc assay showing the effect of LYAR on RNAP I–dependent transcription. 293T cells were co-transfected with the prHu3-Luc reporter gene (firefly luciferase) and internal control vector pRL-TK (Renilla luciferase) along with the indicated amount of HBF and/or HBF-LYAR expression vectors, and luciferase activities were measured. The graph shows the ratio of the luciferase activities (firefly/Renilla) along with the corresponding HBF-LYAR expression levels or levels of HBF only. Data represent the mean ± SEM of three independent experiments. *P < 0.05, **P < 0.01 (paired t-test). (**G**) Methylation of rDNA in HBF-LYAR-TO cells was assessed with the HpaII resistance assay. HBF-LYAR-TO cells were treated with Dox for 24 h and then subjected to HpaII resistance assay. ChIP-CHOP experiment, in which the rDNA promoter-proximal DNA that had been subjected to ChIP with anti-LYAR or anti-UBF was subjected to HpaII digestion to determine whether LYAR associates with transcriptionally active, unmethylated rDNA repeats.

We previously reported that cancer cells, such as HeLa and MCF7, have reduced ribosome production and proliferation when the level of LYAR is low; when the level of LYAR is elevated, however, cell proliferation increases (20). Because LYAR accelerates the processing of pre-rRNA in cultured cells including HeLa cells, we postulated that LYAR also enhances ribosome production by accelerating rRNA synthesis in tumor cells to help maintain their rapid proliferation. To test this idea, we first examined the association of LYAR with rDNA in cultured cells including HeLa, MCF7, and 293T cells by ChIP in combination with quantitative PCR using primer sets specific for regions corresponding to the rDNA loci H0, H8, H13 and H27 that had been defined by O’Sullivan *et al.* (49) (Figure 1A and B, Supplementary Figure S2A and B). We found that LYAR binds to the promoter region (H0) and transcribed region (H8 to H13) of rDNA but not to the intergenic spacer encompassing H27 in HeLa (Figure 1B), MCF7 (Supplementary Figure S2A), and 293T cells (Supplementary Figure S2B). This association of LYAR with rDNA was reduced upon LYAR knockdown in HeLa (Figure 1B) and MCF7 (Supplementary Figure S2C) cells without affecting UBF binding to rDNA, indicating that LYAR is present at the promoter and transcribed regions of rDNA. To minimize the effect of pre-rRNA processing (20), we next examined a 30-min incorporation of 4-thiouridine in 47S/45S pre-rRNA prepared from cells treated with one of two different short interfering RNAs (siRNA), and showed that it was reduced to 40–60% compared with that measured in cells treated with negative-control RNA (ncRNA) (Figure 1C).

To examine the effects of LYAR overexpression on its binding to rDNA and on 47S/45S pre-rRNA synthesis, we produced a doxycycline (Dox)-inducible Flp-In T-REx 293 cell line expressing HBF (His6-biotinylation sequence-FLAG tag)-LYAR (HBF-LYAR-TO cells). The expression of HBF-LYAR increased gradually over time after the addition of Dox, reaching a steady state at a level 10-fold greater than that of endogenous LYAR (Supplementary Figure S2D). The induced HBF-LYAR localized to the nucleolus, as did endogenous LYAR, based on immunocytochemistry (Supplementary Figure S2E). The upregulated expression of HBF-LYAR led to an increase in HBF-LYAR association with the promoter and transcribed region of rDNA (Figure 1D) and promoted the synthesis of 47S/45S pre-rRNA compared with noninduced cells (Figure 1E). In addition, the increased transcription of rDNA upon HBF-LYAR overexpression was demonstrated with a luciferase assay using the prHu3-Luc reporter gene fused in tandem with a rDNA promoter (Figure 1F). Notably, overexpression of HBF-LYAR did not alter the DNA methylation status at rDNA loci, although HBF-LYAR indeed localized to regions of activated, unmethylated rDNA, as did UBF (Figure 1G). Collectively, these data suggested that LYAR promotes rDNA transcription without activating silent rDNA.

### LYAR associates with a number of proteins involved in transcription

**Figure 2.**
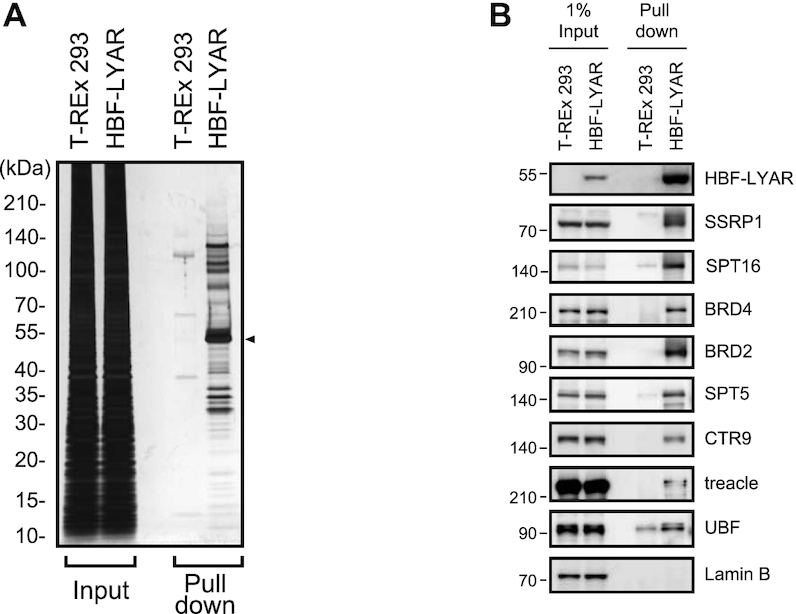
Pulldown of LYAR and identification of LYAR-associated proteins (**A**) Silver staining of HBF-LYAR-associated proteins. HBF-LYAR-associated complexes were isolated *via* sequential two-step pulldown (Ni-NTA pulldown, RNase A treatment, and pulldown of FLAG-tagged HBF-LYAR) from nuclear extract of HBF-LYAR-TO cells or T-REx 293 cells (control) treated with Dox for 24 h. Proteins were subjected to SDS-PAGE and visualized with silver staining. The arrowhead represents HBF-LYAR, as the bait protein. Molecular mass markers (kDa) are indicated to the left. Input: nuclear extract (10 μg). (**B**) Immunoblotting of HBF-LYAR-associated proteins using antibodies indicated to the right of each panel. 1% Input: 1% of the nuclear extract used for pull down of HBF-LYAR complexes. HBF-LYAR was detected by Stabilized Streptavidin-HRP Conjugate.

To gain insight into the role of LYAR in rDNA transcription, we isolated LYAR-associated proteins present in the nucleus. Because exogenously expressed HBF-LYAR was indistinguishable from endogenous LYAR in terms of the elution profile of the sucrose gradient ultracentrifugation of the nuclear extract (Supplementary Figure S3A), we prepared a nuclear fraction containing UBF (Nuc in Supplementary Figure S3B) from HBF-LYAR-TO cells and isolated HBF-LYAR-associated complexes from the Nuc fraction with a two-step purification using His and FLAG tags after RNase A treatment (Figure 2A), which did not affect the interaction between LYAR and nucleolin (Supplementary Figure S3C) as reported (23). The purified HBF-LYAR-associated complexes were separated by SDS-PAGE. The gel was then cut into four pieces, each of which was subjected to in-gel digestion with trypsin and endopeptidase Lys-C. The resulting peptides were analyzed with a nanoscale liquid chromatography–coupled tandem mass spectrometry (LC-MS/MS) system (50,51). These proteomic analyses assigned 148 proteins in total, including those known to associate with LYAR, i.e., nucleophosmin/B23, nucleolin, DDX21, and treacle (Table 1, Supplementary Tables S3 and S4). Among those, seven proteins, namely SSRP1, SPT16, BRD4, BRD2, SPT5, CTR9 and treacle, were also detected by immunoblotting (Figure 2B). Although UBF was identified only occasionally during our MS analyses (not shown in Supplementary Tables S3 and S4, and Table 1), it was detected unambiguously by immunoblotting (Figure 2B). In part, this may have been a consequence of the lower sensitivity of the MS-based method compared with immunoblotting and partly because of the tight binding of UBF to rDNA, which results in low extraction efficiency. As a negative control, lamin B was not detected by MS or immunoblotting (Figure 2B). The identified proteins were classified in Gene Ontology terms that were assigned using DAVID (http://www.geneontology.org/). The top three most abundant proteins had Gene Ontology terms for transcription (under Biological process) and those related to protein-binding, poly(A) RNA binding, and DNA binding (under Molecular function) (Supplementary Figure S3D and Supplementary Table S5). Among the identified proteins, nine are components of three elongation complexes involved in rDNA transcription, i.e., the PAF1 complex (RTF1, CTR9, LEO1, WDR61, PAF1) (13,52), FACT complex (SPT16, SSRP1) (12,53,54), and DSIF complex (SPT4, SPT5) (55,56) (Table 1). The other proteins, namely CD11B, CSK22, CSK2B, PELP1, MAX, treacle, TOP1, TOP2 and XRCC5, also participate in rDNA transcription (3) (Table 1). In addition, nucleophosmin and nucleolin participate in rDNA transcription as histone chaperones (Table 1) (14,57). Collectively, these analyses suggested that LYAR is a component of certain complexes that mediate rDNA transcription.

**Table 1.** LYAR-associating proteins involved or expected to be involved in transcription

**Entry name**	**Protein name**	**Gene symbol**	**Gene ID**	**Note**
**Transcription-FACT complex**				
SP16H_HUMAN	FACT complex subunit SPT16	SUPT16H	11198	involved in rDNA transcription
SSRP1_HUMAN	FACT complex subunit SSRP1	SSRP1	6749	involved in rDNA transcription
**Transcription-DSIF complex**				
SPT5H_HUMAN	Transcription elongation factor SPT5	SUPT5H	6829	involved in rDNA transcription
SPT4H_HUMAN	Transcription elongation factor SPT4	SUPT4H1	6827	involved in rDNA transcription
**Transcription-PAF1 complex**				
RTF1_HUMAN	RNA polymerase-associated protein RTF1 homolog	RTF1	23168	involved in rDNA transcription
CTR9_HUMAN	RNA polymerase-associated protein CTR9 homolog	CTR9	9646	involved in rDNA transcription
LEO1_HUMAN	RNA polymerase-associated protein LEO1	LEO1	123169	involved in rDNA transcription
WDR61_HUMAN	WD repeat-containing protein 61	WDR61	80349	involved in rDNA transcription
PAF1_HUMAN	RNA polymerase II-associated factor 1 homolog	PAF1	54623	involved in rDNA transcription
**Transcription-Super elongation complex (SEC)**				
AFF4_HUMAN	AF4/FMR2 family member 4	AFF4	27125	
ENL_HUMAN	Protein ENL	MLLT1	4298	
**Kinase**				
CD11B_HUMAN	Cyclin-dependent kinase 11B	CDK11B	984	
CSK22_HUMAN	Casein kinase II subunit alpha	CSNK2A2	1459	
CSK2B_HUMAN	Casein kinase II subunit beta	CSNK2B	1460	
**Topoisomerase & DNA damage**				
TOP1_HUMAN	DNA topoisomerase 1	TOP1	7150	involved in rDNA transcription
DDB1_HUMAN	DNA damage-binding protein 1	DDB1	1642	
TOP2A_HUMAN	DNA topoisomerase 2-alpha	TOP2A	7153	involved in rDNA transcription
XRCC5_HUMAN	X-ray repair cross-complementing protein 5	XRCC5	7520	involved in rDNA transcription
**Transcription-Other**				
SAFB2_HUMAN	Scaffold attachment factor B2	SAFB2	9667	
SAFB1_HUMAN	Scaffold attachment factor B1	SAFB	6294	
BRD4_HUMAN	Bromodomain-containing protein 4	BRD4	23476	
BRD2_HUMAN	Bromodomain-containing protein 2	BRD2	6046	
BRD3_HUMAN	Bromodomain-containing protein 3	BRD3	8019	
CN166_HUMAN	UPF0568 protein C14orf166	C14orf166	51637	
PELP1_HUMAN	Proline-, glutamic acid- and leucine-rich protein 1	PELP1	27043	involved in rDNA transcription
MATR3_HUMAN	Matrin-3	MATR3	9782	
RBP56_HUMAN	TATA-binding protein-associated factor 2N	TAF15	8148	
T2FA_HUMAN	General transcription factor IIF subunit 1	GTF2F1	2962	
BCLF1_HUMAN	Bcl-2-associated transcription factor 1	BCLAF1	9774	
YLPM1_HUMAN	YLP motif-containing protein 1	YLPM1	56252	
TCOF_HUMAN	Treacle protein	TCOF1	6949	involved in rDNA transcription
IWS1_HUMAN	Protein IWS1 homolog	IWS1	55677	
SPT6H_HUMAN	Transcription elongation factor SPT6	SUPT6H	6830	
MTA1_HUMAN	Metastasis-associated protein MTA1	MTA1	9112	
SATB2_HUMAN	DNA-binding protein SATB2	SATB2	23314	
MAX_HUMAN	Protein max	MAX	4149	involved in rDNA transcription
SLTM_HUMAN	SAFB-like transcription modulator	SLTM	79811	
TCF20_HUMAN	Transcription factor 20	TCF20	6942	
ILF3_HUMAN	Interleukin enhancer-binding factor 3	ILF3	3609	
S30BP_HUMAN	SAP30-binding protein	SAP30BP	29115	

### UBF is involved in recruitment of LYAR to rDNA

Given that LYAR binds the DNA sequence GGTTAT through its zinc-finger domain that is similar to the one found in the globin gene (21), we first examined whether LYAR interacts directly with rDNA *via* the GGTTAT sequence that is located in five different regions of rDNA (GenBank, U13369.1): nucleotides 42992–42997 (promoter), 3955–3960 (complementary strand, transcribed region), 32632–32637 and 34176–34181 (complementary strand, intergenic spacer), and 37256–37261 (intergenic spacer). However, the locations of these LYAR-binding sequences were not consistent with the regions of rDNA bound to LYAR observed by ChIP analysis; i.e., LYAR was not present in intergenic spacer. In fact, an H0-specific oligonucleotide containing the 42992–42997 sequence present in the promoter region did not bind LYAR as determined with electrophoretic mobility shift assay (Supplementary Figure S4A). To further examine the involvement, if any, of the LYAR zinc-finger domain in rDNA binding, we constructed domain mutants of LYAR, including ones that lack the N-terminal zinc-finger domain (LYAR_168–379_ and LYAR_168–260_; Supplementary Figure S4B) and found that LYAR_168–379_ could minimally bind rDNA (Supplementary Figure S4C). Collectively, these data suggested that the zinc-finger domain of LYAR and LYAR-binding sequences in rDNA are not involved in LYAR binding to rDNA. Because a LYAR mutant lacking the zinc-finger domain has a dominant-negative effect on the processing of pre-rRNAs (20), the zinc-finger domain of LYAR probably participates in pre-rRNAs processing rather than rDNA transcription.

**Figure 3.**
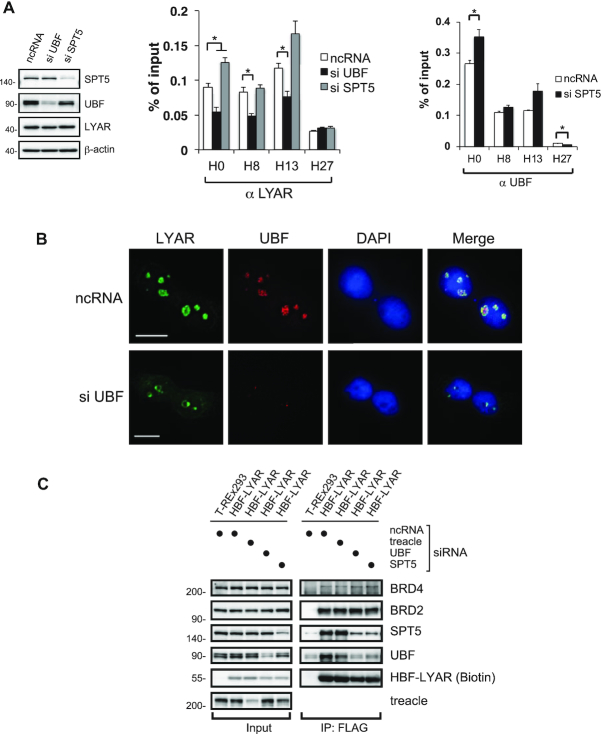
LYAR recruitment to rDNA loci depends on UBF (**A**) ChIP analysis of LYAR (left) or UBF binding (right) to rDNA loci in 293T cells upon knockdown of UBF or SPT5. 293T cells were treated with an siRNA specific for UBF or SPT5 (ncRNA as a control) for 72 h, and whole-cell extract was subjected to immunoblotting using the antibodies indicated to the right of the panels. β-actin was used as the loading control. Molecular mass markers (kDa) are indicated to the left. The graphs show the amount of ChIPed DNA (% of input) with respect to particular rDNA loci. Data represent the mean ± SEM of three independent experiments. *P < 0.05 (unpaired *t*-test). (**B**) Immunocytostaining of LYAR and UBF upon UBF knockdown. 293T cells were treated with ncRNA or an siRNA specific for UBF for 72 h and subjected to immunocytostaining with anti-LYAR (rabbit) and anti-UBF (mouse). FITC-conjugated anti-rabbit IgG and Cy3-conjugated anti-mouse IgG were used as the secondary antibodies. DAPI staining indicates the nucleus. Bar: 10 μm. (**C**) Immunoblotting for HBF-LYAR-associated proteins upon siRNA-mediated knockdown of treacle, UBF or SPT5 in HBF-LYAR-TO cells for 72 h. A two-step pulldown was carried out with His6- or FLAG-tag of HBF-LYAR. The antibodies used for immunoblotting are indicated to the right.

We next considered whether LYAR binds rDNA loci *via* the basal pre-transcription structural proteins UBF and treacle (58–67), each of which can associate with LYAR; this analysis was carried out in concert with knockdown of UBF, treacle or SPT5 (as a control). We found that UBF is required for the recruitment of LYAR to regions H0, H8, and H13 of rDNA as shown by ChIP analysis of UBF-knockdown cells (Figure 3A), whereas treacle seems to contribute to the recruitment of LYAR to other regions in rDNA (H0 and H8) (Supplementary Figure S4D). In addition, UBF knockdown reduced the localization of LYAR in the nucleolus and shifted to the nucleolar cap (Figure 3B). Given our previous report that LYAR knockdown did not alter the nucleolar localization of UBF (20), these data suggested that LYAR is recruited to rDNA *via* UBF. We expected that the knockdown of SPT5 would not affect LYAR binding to rDNA; however, it significantly increased LYAR binding to region H0 of rDNA (Figure 3A). This increase coincided with the increase of UBF binding to the H0 region upon SPT5 knockdown (Figure 3A). These data suggested that LYAR is recruited to rDNA *via* UBF and that SPT5 can control LYAR binding to region H0. On the other hand, the knockdown of UBF or SPT5 reduced the binding of LYAR to SPT5 or UBF, respectively (Figure 3C), suggesting that LYAR can form a complex with UBF and SPT5. These results could not definitively determine whether the LYAR-UBF-SPT5 complex forms upon binding to rDNA or forms prior to binding, i.e. as a pre-formed unit that might help maintain the equilibrium among the three protein monomers. Therefore, the mechanism by which UBF and SPT5 regulate LYAR recruitment to rDNA may be more complicated than previously suspected.

Given that the small molecule CX-5461 inhibits RNAP I transcription initiation *via* exclusion of SL1 from the rDNA promoter and that actinomycin D (actD) inhibits the elongation of rDNA transcription (68,69), we examined the effects of these compounds on the binding of UBF and LYAR to rDNA. Although both compounds inhibited rRNA synthesis (Supplementary Figure S4E), they did not affect the binding of LYAR to UBF at 6 h treatment (Supplementary Figure S4E and F), suggesting that these two proteins form a complex independently of transcription. However, actD reduced the binding between LYAR and UBF at 2 h treatment and increased the binding of LYAR to the transcribed region of rDNA but not the promoter or the intergenic spacer (Supplementary Figure S4G). Thus, the inhibition of rDNA transcriptional elongation seems to rearrange the binding between LYAR and UBF and to trap LYAR on the transcribed region of rDNA.

### LYAR recruits BRD2 to rDNA loci

**Figure 4.**
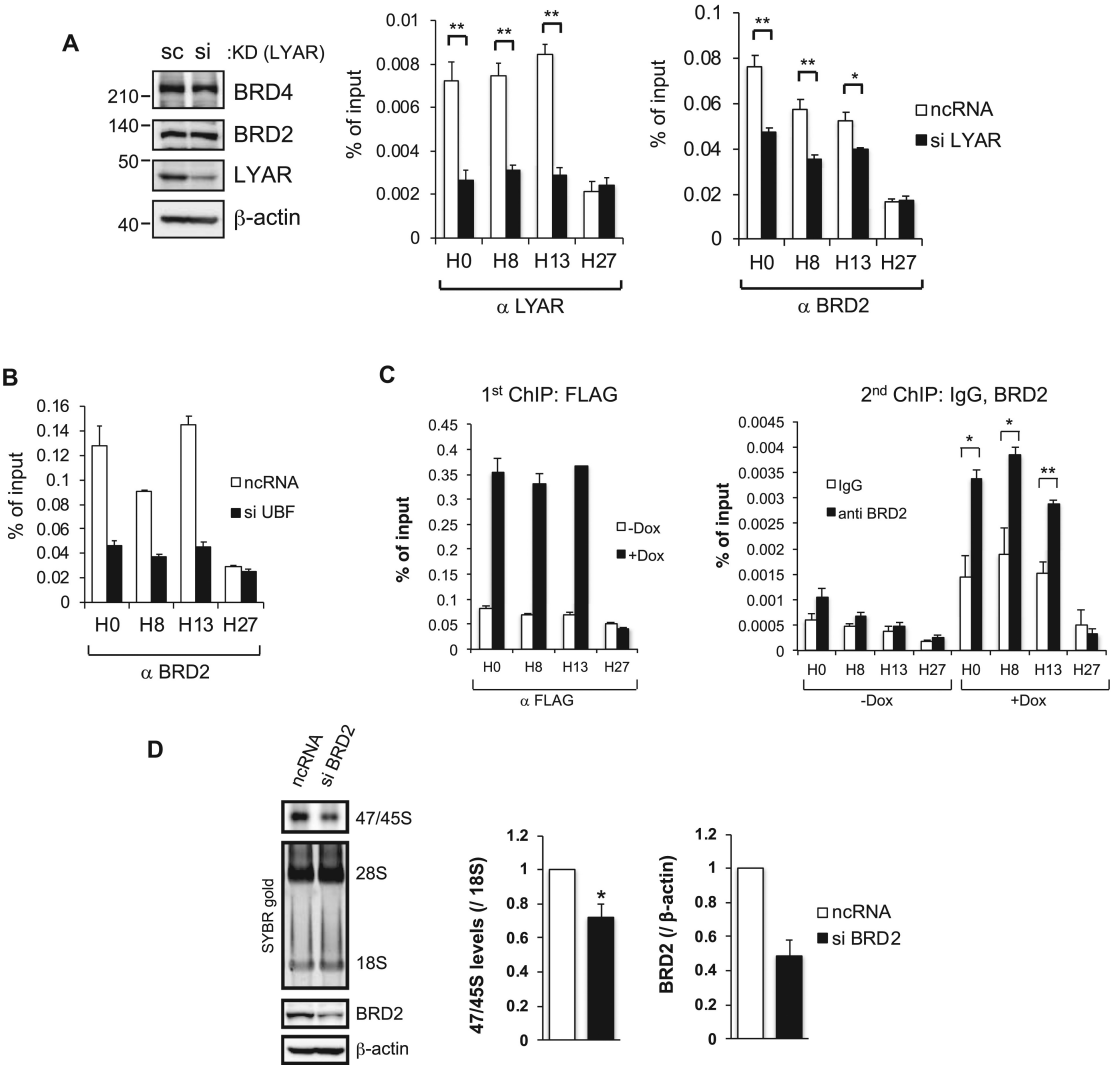
LYAR recruits BRD2 to rDNA transcription sites (**A**) ChIP analysis of BRD2 binding to rDNA loci in 293T cells upon LYAR knockdown (KD). 293T cells were treated with an siRNA specific for LYAR (or ncRNA, control) and then subjected to ChIP analysis with an antibody against LYAR or BRD2. LYAR KD was confirmed by immunoblotting with antibodies indicated to the right of the panels. β-actin was used as the loading control. Molecular mass markers (kDa) are indicated to the left of the panels. The graphs show the amount of ChIPed DNA (% of input) relative to the number of rDNA loci with the antibody indicated under each graph. Data represent the mean ± SEM of three independent experiments. *P < 0.05, **P < 0.01 (unpaired *t*-test). (**B**) ChIP analysis of BRD2 binding to rDNA loci in 293T cells upon UBF knockdown. The cells were treated with an siRNA specific for UBF (or ncRNA) and then subjected to ChIP analysis with an antibody against BRD2. The graph shows the amount of ChIPed DNA (% of input). (**C**) Re-ChIP analysis showing that LYAR-BRD2-associated complexes bind rDNA loci. The first ChIP of HBF-LYAR, with anti-FLAG, was performed using HBF-LYAR-TO cells with or without Dox treatment (1^st^ ChIP; left graph). The second ChIP, with anti-BRD2, was performed using the first ChIPed HBF-LYAR-associated complexes (2^nd^ ChIP; right graph). As an antibody control for anti-BRD2, a nonspecific rabbit IgG was used. The graphs show the amount of ChIPed DNA (% of input). Data represent the mean ± SEM of three independent experiments. *P < 0.05, **P < 0.01 (unpaired *t*-test). (**D**) Metabolic labeling (4-thiouridine) of newly synthesized 47/45S pre-rRNA in 293T cells upon BRD2 knockdown (siRNA). The pre-rRNA was biotinylated and then subjected to agarose gel electrophoresis under denaturing condition and northern blotting. The signals for 47/45S pre-rRNA were detected by chemiluminescence. 28S and 18S rRNAs were used as loading controls (stained with SYBR gold). The graph shows the relative band intensities of biotin-labeled 47/45S pre-rRNA normalized to that of 18S rRNA. Data represent the mean ± SEM of four independent experiments. *P < 0.05, **P < 0.01 (paired *t*-test). Knockdown of BRD2 was confirmed by immunoblotting with anti-BRD2. β-actin was used as the loading control.

Given that we identified a number of transcription regulatory factors as LYAR-binding proteins (Table 1), we investigated which transcription regulatory factors have to be recruited to rDNA to increase the transcription of 47S/45S pre-rRNA *via* binding to LYAR and looked for transcription regulatory factors for which the binding to rDNA is reduced upon siRNA-mediated knockdown of LYAR. Among the transcription regulatory factors we examined (Supplementary Figure S5A), BRD2 exhibited reduced binding to rDNA (regions H0, H8 and H13) upon LYAR knockdown, although the overall expression of BRD2 did not change (Figure 4A). Knockdown of BRD2 itself reduced its binding to rDNA, including the region corresponding to H27 (Supplementary Figure S5B), implying that BRD2 is recruited at a low basal level to rDNA loci in the absence of LYAR. The binding of LYAR to BRD2 was not affected by the inhibition of RNAP I transcription with actD or CX-5461 (Supplementary Figure S4F), suggesting that the binding of LYAR to BRD2 is independent on the RNAP I transcription. By contrast, knockdown of UBF reduced the binding of BRD2 in regions H0, H18, and H13 but not H27, as did LYAR (Figure 4A and B). Collectively, these data strongly suggested that LYAR enhances the recruitment of BRD2 to the promoter and transcribed regions of rDNA *via* UBF.

Given our result that the peptide LYAR_168–260_, which is rich in lysine residues, could interact with BRD2 (Supplementary Figure S4B), we examined *in vitro* binding between LYAR_168–260_ and each of the recombinant domain mutants of BRD2 (Supplementary Figure S5C). LYAR_168–260_ could interact with BRD2_458–720_ or BRD2_458–801_, each of which lacks the two canonical bromodomains (Supplementary Figure S5D). Isothermal titration calorimetry also revealed that peptide BRD2_489–540_ could interact with peptide LYAR_175–219_ (*K*_d_ = 12.3 µM) with 1:1 stoichiometry (Supplementary Figure S5E). These results were consistent with recent reports by Luna-Pelaez *et al.* showing that the motif B region of BRD2 interacts with the C-terminal region of LYAR (70). In addition, overexpression of LYAR increased the binding of BRD2 to rDNA loci without affecting the overall cellular level of BRD2 (Supplementary Figure S5F). Sequential re-ChIP analysis revealed co-occupancy of LYAR and BRD2 in rDNA regions H0, H8 and H13 (Figure 4C), supporting the idea that LYAR forms a complex with BRD2 *via* direct interaction and then the complex associates with rDNA loci *via* UBF. The fact that BRD2 overexpression did not affect the binding of LYAR or UBF to rDNA (Supplementary Figure S5G) excluded the possibility that BRD2 could increase the binding of LYAR or UBF to rDNA loci. Because BRD proteins had not previously been shown to be involved in rDNA transcription, we then knocked down BRD2 and found that 47S/45S pre-rRNA synthesis was reduced to the level of ∼60% compared with the mock-knockdown control, as detected by a 30-min incorporation of 4-thiouridine (Figure 4D). Collectively, these data suggested that BRD2 is required for LYAR-dependent rRNA synthesis.

### BRD2 recruits KAT7 to rDNA loci

**Figure 5.**
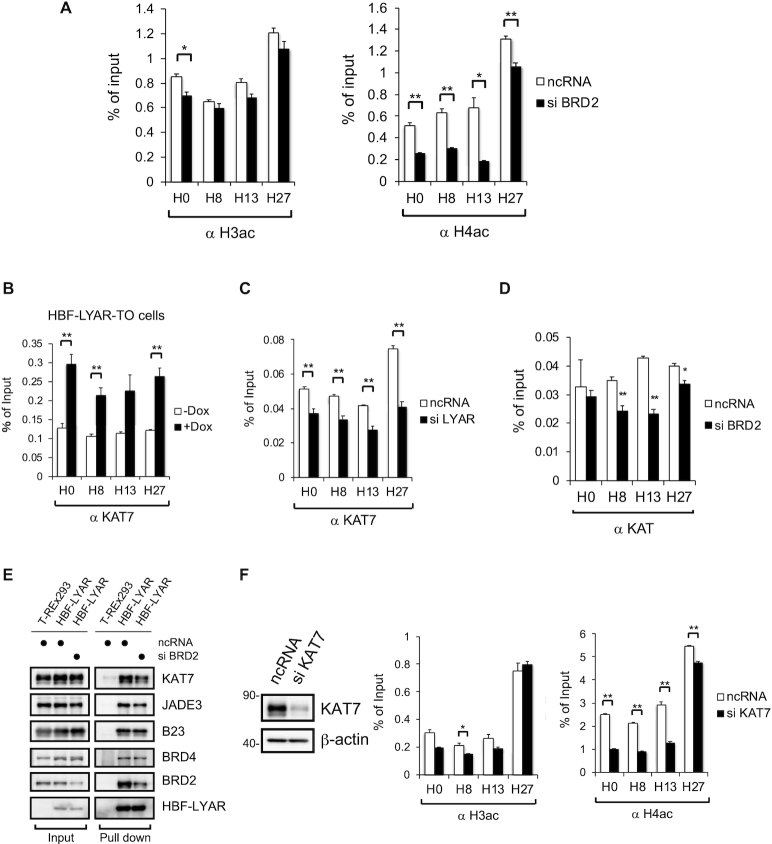
BRD2 recruits KAT7 to rDNA loci (**A**) ChIP analysis of the extent to which H3 or H4 is acetylated. 293T cells were treated with an siRNA specific for BRD2 or ncRNA (control) and then subjected to ChIP analysis with an antibody against H3ac or H4ac, as indicated. The graphs show the amount of ChIPed DNA (% of input) relative to the number of rDNA loci indicated under each graph. Data represent the mean ± SEM of three independent experiments. *P < 0.05, **P < 0.01 (unpaired t-test). B–D) ChIP analysis of the binding of KAT7 to rDNA loci. HBF-LYAR-TO cells were treated with (+Dox) or without (–Dox) Dox (**B**). 293T cells were treated with ncRNA (control) or an siRNA specific for LYAR (**C**) or BRD2 (**D**) for 72 h. These cells were subjected to ChIP analysis with an antibody against KAT7. The graphs show the amount of ChIPed DNA (% of input) relative to the number of rDNA loci indicated under each graph. Data represent the mean ± SEM of three independent experiments. *P < 0.05, **P < 0.01 (unpaired t-test). (**E**) Immunoblotting for HBF-LYAR-associated proteins upon the knockdown of BRD2. HBF-LYAR-TO cells were treated with an siRNA specific for BRD2 for 72 h; after induction with Dox, the nuclear extract was subjected to the pulldown with two-step pull down with His6- and FLAG-tag of HBF-LYAR. HBF-LYAR-associated proteins were detected by immunoblotting with antibodies indicated to the right of the panels. (**F**) ChIP analysis of the extent to which H3 or H4 is acetylated. 293T cells were treated with an siRNA specific for KAT7. These cells were subjected to ChIP analysis with an antibody against H3ac or H4ac, as indicated. 293T cells were treated with ncRNA (control) or an siRNA specific for KAT7. The graphs show the amount of ChIPed DNA (% of input) relative to the number of rDNA loci indicated under each graph. Data represent the mean ± SEM of three independent experiments. *P < 0.05, **P < 0.01 (unpaired t-test).

We further addressed the issue of how BRD2 can upregulate LYAR-dependent rDNA transcription. Given that BRD2 recruits the histone H4–specific acetyltransferase to the RNAP II transcription start site (43,71,72), we first examined whether LYAR-recruited BRD2 could induce acetylation of local histones at rDNA loci by using ChIP with antibodies that recognize acetylated H3 (at K9, K14, K18, K23 and K27) or acetylated H4 (at K5, K8, K12 and K16). Similar to the report by Sinha *et al.* (43), BRD2 knockdown mainly reduced H4 acetylation at rDNA loci (Figure 5A). Based on our MS-based identification of two LYAR-associated histone acetyltransferases, namely KAT7 (45) and BRD4 (44) (Table 1 and Supplementary Table S3), we first examined whether KAT7 is responsible for the acetylation of histones at rDNA loci. We found that LYAR overexpression increased the binding of KAT7 to rDNA, including the intergenic spacer region corresponding to the probe for region H27 (Figure 5B). By contrast, LYAR knockdown reduced KAT7 binding to rDNA (Figure 5C). These data demonstrated that LYAR has an effect on KAT7 binding to rDNA beyond the LYAR-binding region. Moreover, knockdown of BRD2 reduced the amount of rDNA-associated KAT7 at regions H8, H13 an H27 (Figure 5D). This BRD2 knockdown also reduced the binding of LYAR to KAT7 and JADE3 that was identified as one of the LYAR-binding proteins, a possible scaffold partner of KAT7 (Figure 5E), suggesting that BRD2 bridges between LYAR and KAT7 (or more likely between LYAR and KAT7-JADE3). In addition, KAT7 knockdown mainly reduced the acetylation of histone H4 at rDNA loci (Figure 5F). Collectively, these data suggested that LYAR recruits BRD2-KAT7 and possibly JADE3 to acetylate histone H4 within nucleosomes at rDNA loci.

### BRD2 assists the recruitment of BRD4 to rDNA loci

**Figure 6.**
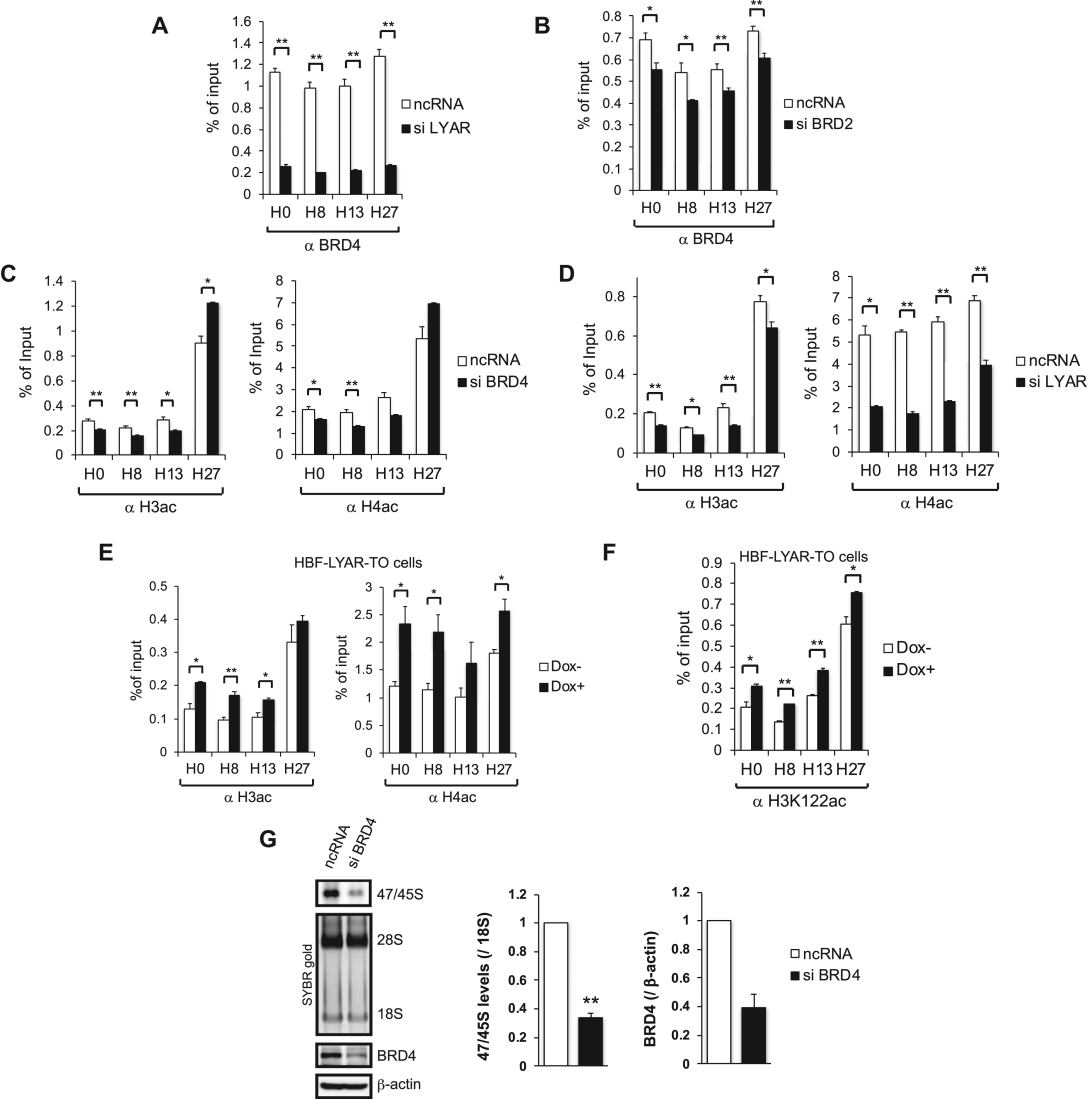
LYAR and BRD2 assist the recruitment of BRD4 to rDNA (**A, B**) ChIP analysis of the binding of BRD4 to rDNA upon the knockdown of LYAR (A) or BRD2 (B). 293T cells were treated with ncRNA or an siRNA specific for LYAR (A) or BRD2 (B) for 72 h. These cells were subjected to ChIP analysis with an antibody against BRD4. The graphs show the amount of ChIPed DNA (% of input) relative to the number of rDNA loci indicated under each graph. Data represent the mean ± SEM of three independent experiments. *P < 0.05, **P < 0.01 (unpaired t-test). (**C, D**) ChIP analysis of the extent to which H3 or H4 is acetylated in 293T cells. The cells were treated with ncRNA or an siRNA specific for BRD4 (C) or LYAR (D) for 72 h. These cells were subjected to ChIP analysis with an antibody against H3ac or H4ac, as indicated. (**E, F**) ChIP analysis of the extent to which H3 or H4 is acetylated in HBF-LYAR-TO cells. The cells were treated with (Dox+) or without Dox (Dox–) for 24 h. These cells were subjected to ChIP analysis with an antibody against H3ac or H4ac (E), or H3K122ac (F). (**G**) Metabolic labeling (4-thiouridine) of newly synthesized 47/45S pre-rRNA in 293T cells upon BRD4 knockdown (siRNA) for 72 h. The pre-rRNA was biotinylated and then subjected to agarose gel electrophoresis and northern blotting. The signals for 47/45S pre-rRNA were detected by chemiluminescence. 28S and 18S rRNAs were used as loading controls (stained with SYBR gold). The graph shows the relative band intensities of biotin-labeled 47/45S pre-rRNA normalized to that of 18S rRNA. Data represent the mean ± SEM of four independent experiments. *P < 0.05, **P < 0.01 (paired *t*-test). Knockdown of BRD4 was confirmed by immunoblotting with anti-BRD4. β-actin was used as the loading control.

We next examined the contribution of another LYAR-binding acetyltransferase, namely BRD4, to histone acetylation at rDNA loci. The overexpression of LYAR increased the binding of BRD4 to rDNA loci without affecting the overall expression level of BRD4 (Supplementary Figure S5F). In addition, the knockdown of LYAR or BRD2 reduced the binding of BRD4 to rDNA (Figure 6A and B); as expected, knockdown of BRD4 reduced its own association with rDNA loci (Supplementary Figure S6A). BRD4 knockdown reduced the acetylation of both histones H3 and H4 at rDNA loci, except for region H27 (Figure 6C). Moreover, LYAR knockdown reduced the overall acetylation of both H3 and H4 at the rDNA promoter as well as the transcribed and intergenic spacer regions of rDNA loci (Figure 6D), whereas LYAR overexpression increased the overall acetylation of both H3 and H4 (Figure 6E). Given that BRD4 catalyzes the acetylation of H3K122 (H3acK122), which is sufficient to stimulate transcription of rDNA (73), we also tested whether acetylation at H3K122 is increased by overexpression of LYAR *via* the ability to recruit BRD4 to rDNA. ChIP analysis with anti-H3acK122 revealed that LYAR overexpression caused a significant increase in H3acK122 at rDNA loci including region H27 (Figure 6F), suggesting that LYAR-recruited BRD4 is also responsible for H3K122 acetylation at rDNA loci. Consistent with these data, BRD4 knockdown reduced 47S/45S pre-rRNA synthesis to ∼35% compared with the mock-knockdown control, as detected by a 30-min incorporation of 4-thiouridine (Figure 6G). However, the histone acetyltransferases GCN5 and p300 were not identified as LYAR-associated proteins (Supplementary Tables S3 and S4), and indeed LYAR did not affect the binding of either GCN5 or p300 to rDNA (Supplementary Figure S6B) (11,74). Collectively, these data suggested that LYAR-mediated recruitment of BRD2, KAT7 and BRD4 to rDNA controls the acetylation of local histones H3 and H4 to cause the relaxation of these histones from rDNA loci and thereby promote rDNA transcription.

### BRD2 and BRD4 form complexes with LYAR and KAT7-JADE3 independently of each other

**Figure 7.**
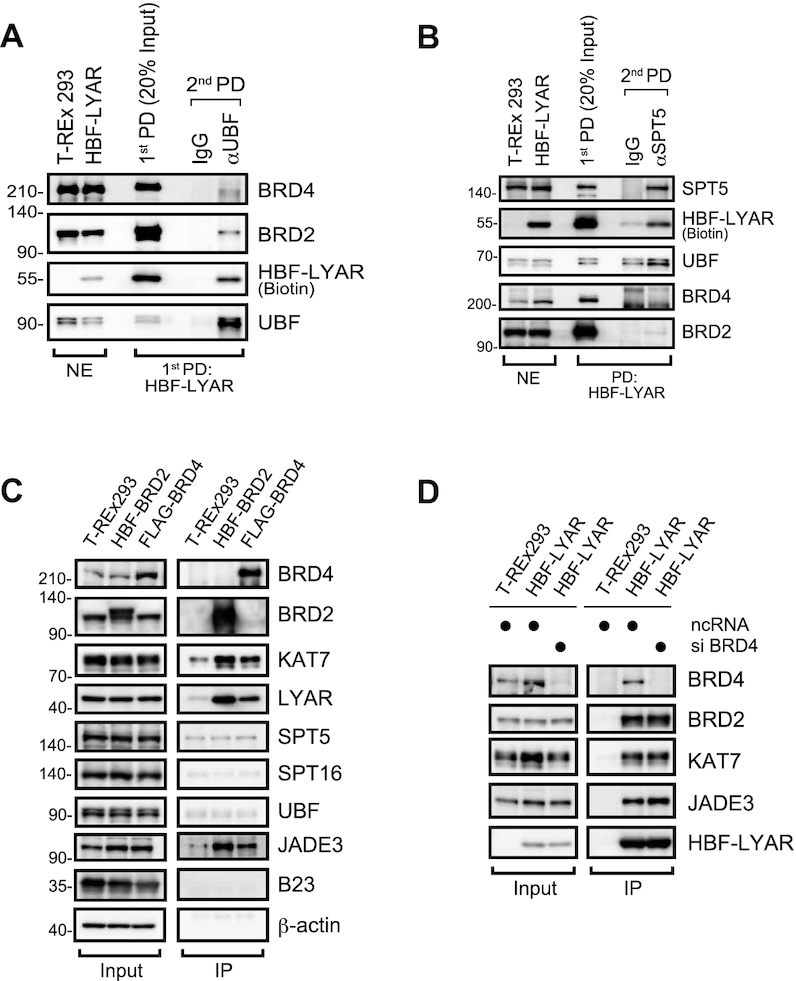
BRD2, BRD4, and SPT5 bind LYAR independently (**A, B**) Reverse pulldown (PD) of UBF (A) or SPT5 (B) from HBF-LYAR complexes, assessed with immunoblotting. HBF-LYAR-TO cells were treated with Dox for 24 h, and then nuclear extracts (NE) were subjected to first pulldown (first PD) by two-step immunoprecipitation using His6- and FLAG-tag of HBF-LYAR. The purified HBF-LYAR complexes were immunoprecipitated with anti-UBF (αUBF) (A) or anti-SPT5 (αSPT5) (B) as the second affinity bait (second PD). T-REx 293 cells treated with Dox were used as the control. Proteins were detected by immunoblotting using the antibodies indicated to the right of the panels. Molecular mass markers (kDa) are indicated to the left of the panels. The nuclear extract (NE, 10 μg) was used as a loading control. (**C**) Immunoblotting for proteins that associated with BRD2 or BRD4. HBF-BRD2-TO cells and FLAG-BRD4-TO cells were treated with Dox for 24 h, and then the nuclear extracts were subjected to a two-step immunoprecipitation (IP) with anti-His6 and anti-FLAG. T-REx 293 cells treated with Dox were used as the control. Proteins that associated with HBF-BRD2 or FLAG-BRD4 were detected by immunoblotting using the antibodies indicated to the right of the panels. β-actin was used as the loading control. (**D**) Immunoblotting for HBF-LYAR-associated proteins upon the knockdown of BRD4. HBF-LYAR-TO cells were treated with an siRNA specific for BRD4 for 72 h. After induction with Dox, the nuclear extracts were subjected to two-step immunoprecipitation (IP) using His6- and FLAG-tag of HBF-LYAR.

To examine the relationship among complexes formed by LYAR, UBF, BRD2/4, KAT7-JADE3 and SPT5, we carried out a reverse pulldown assay using each of those proteins, individually, as the affinity bait. We initially performed a two-step pulldown using HBF-LYAR as the first affinity bait and endogenous SPT5 or UBF as the second affinity bait. Although LYAR-UBF could associate with BRD2 or BRD4 even at basal cellular levels (Figure 7A), LYAR-SPT5 did not associate with BRD2 or BRD4 at all (Figure 7B). Conversely, a pulldown assay using BRD2 or BRD4 as bait showed that neither protein could associate with SPT5 or UBF; however, BRD2 and BRD4—independently of each other—could form a complex containing both LYAR and KAT7-JADE3 (Figure 7C). In addition, knockdown of BRD2 did not affect the binding of BRD4 to LYAR, and *vice versa* (Figures 5E and 7D). Collectively, these data support the previous notice that LYAR forms a complex with BRD2 or BRD4, after which either complex can be recruited to rDNA *via* UBF. Furthermore, the association of LYAR with KAT7-JADE3 is dependent on BRD2 (Figure 5E) but not on BRD4 (Figure 7D), suggesting that BRD2 and BRD4 differentially promote the recruitment of KAT7-JADE3 to rDNA.

## DISCUSSION

Our results demonstrate that LYAR has a role in rDNA transcription. Given that LYAR overexpression does not affect the cellular levels of MYC, PTEN, retinoblastoma, and p53 (20), we hypothesized that LYAR promotes rDNA transcription *via* a pathway independent of those proteins, although we could not exclude the possibility that LYAR-induced proteins other than those listed above may be involved in rDNA transcription. Among several transcription regulatory factors that associate with LYAR (Table 1), we identified BRD2, KAT7, and BRD4 as being recruited by LYAR to rDNA. Although BET family proteins and MYST-type acetyltransferases are essential mediators of RNAP II–dependent transcription (75,76), their involvement in RNAP I–dependent transcription is unknown. Thus, to our knowledge, this is the first report that provides evidence of a role for BRD proteins and KAT7 in RNAP I–dependent transcription.

**Figure 8.**
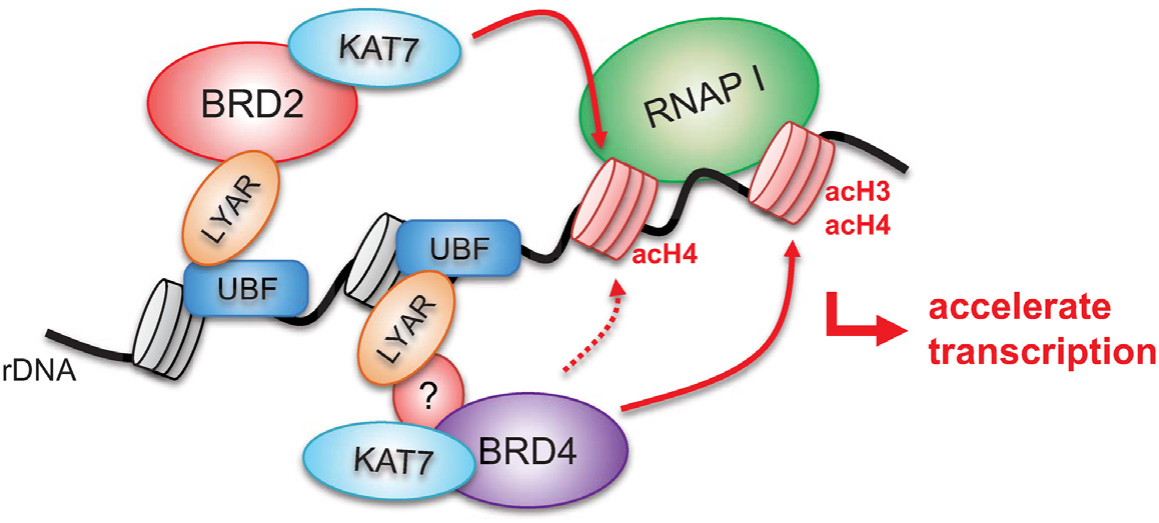
Model for LYAR function in rDNA transcription For RNAP I–dependent transcription, LYAR binds rDNA *via* UBF, recruits BRD2-KAT7-JADE3 through direct binding between LYAR and BRD2 (without any auxiliary factor) to rDNA transcription sites, and accelerates acetylation of histone H4. Moreover, LYAR facilitates the binding of BRD4-KAT7-JADE3 to acetylated H4 at rDNA transcription sites. BRD4-KAT7-JADE3 further accelerates the acetylation of histones H3 and H4 at rDNA loci, which further enhances BRD4-KAT7-JADE3 binding to rDNA loci, resulting in the relaxation of chromatin structure to enhance RNAP I–dependent transcription. Because the binding of LYAR to KAT7-JADE3 is dependent on BRD2 but not BRD4, an unknown factor may be involved in the binding between LYAR and KAT7-JADE3 in the LYAR-BRD4-KAT7-JADE3 complex.

BRD2 associates with the RNAP II mediator complex containing E2F-TATA-box–binding proteins that is proposed to recruit histone acetyltransferases to DNA during RNAP II–dependent transcription (71). However, it is less likely that LYAR recruits the E2F-TATA-box transcription factors to rDNA *via* its association with BRD2 because our MS-based analysis did not identify any of the proteins known to associate with BRD2 (Table 1). Instead of those proteins, we found that BRD2 assists the recruitment of KAT7 and BRD4 to rDNA. Based on our present findings, we propose a mechanism for LYAR-mediated enhancement of rDNA transcription (Figure 8). First, upon its increased expression, LYAR forms a complex with BRD2-KAT7 (or BRD4-KAT7); the complex probably contains JADE3. Second, LYAR-BRD2-KAT7 complex binds to the promoter and transcribed regions of rDNA loci *via* UBF. Third, KAT7 acetylates histone H4 in nucleosomes at rDNA. Alternatively, LYAR may recruit BRD4-KAT7 to rDNA loci regardless of whether BRD2 is already present at those loci to accelerate BRD4-KAT7 binding to acetylated H4. Finally, BRD4-KAT7 assists the acetylation of both H4 and H3 near the LYAR-binding site on rDNA and promotes BRD4-KAT7 binding to rDNA loci to relax the nucleosome structure, which enhances RNAP I–mediated transcription (Figure 8). In this mechanism, LYAR binds directly to BRD2 and recruits it to rDNA loci without involvement of acetylated histones. This is in contrast to the involvement of BRD2 in RNAP II–dependent transcription, in which it is recruited to an acetylated histone (e.g., H4K12ac) (77). By contrast, an unknown protein(s) may be required for the binding of LYAR to BRD4. This proposed mechanism may explain why BRD4 is recruited to the intergenic spacer encompassing H27 beyond region H13 (Figure 6A). A similar function of the MYST-type acetyltransferase has been reported to facilitate HIV (human immunodeficiency virus) latency in human cells; in that case, BRD4 is recruited to the HIV long, terminal repeat by interacting with acetylated H4 (acetylation catalyzed by KAT5) (78).

One issue this mechanism does not address is whether the intrinsic histone acetylase activity of BRD4 is responsible for LYAR-induced acetylation of histones H3 and H4 or whether BRD4 recruits a different acetyltransferase. The lysine residues in H3 and H4 that are acetylated by BRD4 are distinct from those of other histone acetyltransferases, especially the acetylation of K122 in H3 (H3K112ac) (44). Given that overexpression of LYAR increased the frequency of H3acK122 (Figure 6F), it is likely that the intrinsic acetylase activity of BRD4 is involved in acetylation assisted by LYAR on rDNA loci. Although two other acetyltransferases, namely P300/CBP (79) and SIRT7 (80), can generate H3K122ac, we did not identify those acetyltransferases as LYAR-associated proteins, supporting the aforementioned hypothesis that the intrinsic histone acetylase activity of BRD4 is responsible for LYAR-induced acetylation. However, it is possible that other unknown acetyltransferases are involved in LYAR-assisted histone acetylation at rDNA loci.

Another issue with this mechanism is whether LYAR binds rDNA directly or indirectly. Our data show that LYAR binds rDNA indirectly *via* binding to UBF, and we serendipitously found that SPT5 regulates UBF binding to the rDNA promoter, and thus SPT5 probably regulates LYAR binding to that promoter. In addition, we demonstrated that SPT5-UBF and BRD2-KAT7-JADE3 (or BRD4-KAT7-JADE3) form complexes with LYAR independently of each other (Figure 7A–C). Given that UBF and SPT5 participate in the pause and release, respectively, of RNAP I from the rDNA promoter (35,54,81), the initiation of rDNA transcription can be regulated by a similar mechanism, i.e., RNAP I is initially paused at the promoter but is released upon LYAR-BRD2-KAT7-JADE3 binding to UBF. This RNAP I release mechanism is consistent with our result that LYAR affected neither the binding of two RNAP I subunits, RPA194 and RPA135, to rDNA (Supplementary Figure S5A) nor the state of DNA methylation (Supplementary Figure S2F). However, LYAR overexpression did not alter the binding of SPT5 to rDNA loci, including the promoter (Supplementary Figure S5A). Thus, we propose that LYAR binding to rDNA is not involved in the release of paused RNAP I from the promoter, although we cannot exclude the possibility that LYAR binding alters the association between SPT5 and UBF *via* histone acetylation by KAT7/BRD4 at the rDNA promoter or phosphorylation of UBF *via* the kinase activity of BRD2.

Based on our current results and those of our previous study (20), we propose that upregulation of LYAR in certain cancer cells promotes rDNA transcription and pre-rRNA processing, resulting in increased ribosome biogenesis and maintenance of rapid growth and proliferation. Therefore, it will be interesting to determine whether cancer cells that express *LYAR* at a high level can maintain their tumorigenic potential upon suppression of LYAR function in rRNA synthesis. Recent research revealed that the cell-permeable molecule JQ1 inhibits the binding of BRD proteins with acetylated histones and is a potential therapeutic agent for the treatment of acute myeloid leukemia (82,83). Our present findings concerning the potential interactions among LYAR/UBF, LYAR/BRD2-KAT7-JADE3 and/or LYAR/BRD4-KAT7-JADE3 may provide other potential targets for cancer treatment.

Finally, it has been shown that the modulation of rRNA synthesis is closely linked to cell differentiation of ovarian germline stem cells in *Drosophila* (35) and in human cells with differentiation potential, including embryonic stem cells (34) and promyelocytic and monocytic leukemia cells (84). Therefore, it is likely that LYAR maintains the pluripotency of embryonic stem cells by promoting rRNA synthesis. It is also possible that the physiological convergence of BRD2 and BRD4 observed in early embryogenesis in mice (85,86) may be a consequence of their roles—as mediated by LYAR—in rRNA synthesis.

## CLINICAL SPECIMENS

The Ethics Committee of The University of Tokyo approved the use of human tissues, and all patients gave informed consent.

## Supplementary Material

gkz747_Supplemental_Files
