# Supplementary material for: LYAR potentiates rRNA synthesis by recruiting BRD2/4 and the MYST-type acetyltransferase KAT7 to rDNA
Source: Nucleic Acids Res. 2019 Sep 2;47(19):10357–72. doi: 10.1093/nar/gkz747 (PMC6821171; doi:10.1093/nar/gkz747)

SUPPLEMENTARY MATERIALS

LYAR potentiates rRNA synthesis by recruiting BRD2/4 and the MYST-type acetyltransferase KAT7 to rDNA

Keiichi Izumikawa[1,2], Hideaki Ishikawa[1], Harunori Yoshikawa[3], Sally Fujiyama[1], Akira Watanabe[4], Hiroyuki Aburatani[5], Hiroyuki Tachikawa[6], Toshiya Hayano[7], Yutaka Miura[1,2], Toshiaki Isobe[8], Richard J. Simpson[2,9], Li Li[10,11], Jinrong Min[2, 10, 11], and Nobuhiro Takahashi[1,2]§

[1]Department of Applied Life Science, Tokyo University of Agriculture and Technology, 3-5-8 Saiwai-cho, Fuchu-shi, Tokyo 183-8509, Japan
[2]Global Innovation Research Organizations, Tokyo University of Agriculture and Technology, 3-5-8 Saiwai-cho, Fuchu, Tokyo 183-8509, Japan
[3]Centre for Gene Regulation & Expression, School of Life Sciences, University of Dundee, Dow Street, Dundee, DD1 5EH, UK
[4]Department of Life Science Frontiers, Center for iPS Cell Research and Application, Kyoto University 53, Shogoin-kawahara-cho, Sakyo-ku, Kyoto-shi, Kyoto 606-8507, Japan
[5]Laboratory for System Biology and Medicine, University of Tokyo, 4-6-1 Komaba, Meguro-ku, Tokyo 153-8904, Japan
[6]Department of Applied Life Science, The University of Tokyo, 1-1-1 Yayoi, Bunkyo-ku, Tokyo 113-8657, Japan
[7]Department of Biomedical Sciences, College of Life Sciences, Ritsumeikan University, 1-1-1 Nojihigashi, Kusatsu 525-8577, Japan
[8]Department of Chemistry, Graduate School of Sciences and Engineering, Tokyo Metropolitan University, 1-1 Minamiosawa, Hachiouji-shi, Tokyo 192-0397, Japan
[9]La Trobe Institute for Molecular Science (LIMS) LIMS Building 1, Room 412 La Trobe University, Bundoora Victoria 3086, Australia
[10]Structural Genomics Consortium, University of Toronto, 101 College St., Toronto, Ontario, M5G 1L7, Canada
[11]Department of Physiology, University of Toronto, Toronto, Ontario, M5S 1A8, Canada

§To whom correspondence should be addressed: Applied Biological Science, Tokyo University of Agriculture and Technology, 3-5-8 Saiwai-cho, Fuchu, Tokyo 183-8509, Japan. Tel.: +81 042 367 5709; Fax: +81 042 367 5709; E-mail: ntakahas@cc.tuat.ac.jp

Running title: LYAR potentiates rRNA synthesis

Keywords: carcinogenesis / proteomic analysis / cell proliferation / ribosome biogenesis / elongation of rDNA transcription

SUPPLEMENTARY EXPERIMENTAL PROCEDURES

Antibodies and Reagents

Antibodies used in this study are listed in Supplementary Table S1.

Stabilized Streptavidin-HRP Conjugate (Thermo Scientific, 89880D) was used for the detection of biotinylated oligonucleotides or proteins. All general reagents were purchased from Wako Pure Chemical (Osaka, Japan), Kanto Chemical Co. (Tokyo, Japan), or Nacalai Tesque (Kyoto, Japan).

Microarray analysis

The tissue distribution of LYAR mRNA was examined by gene expression analysis with GeneChip U133 plus 2. Microarray data is available in RefExA database (http://www.lsbm.org).

Cell culture

SW480, SW620, HeLa, MCF7, 293T (HEK293 cells transformed with large T antigen) and Flp-In T-REx 293 (T-REx 293) cells were cultured in Dulbecco's modified Eagle's medium (DMEM; Sigma-Aldrich) supplemented with 10% heat-inactivated fetal bovine serum (Biowest LLC) and streptomycin (0.1 mg/ml; Wako) and penicillin G (100 U/ml, Wako) at 37 °C under 5 % CO_2 in air.

Immunoblot analysis

Immunoblot analysis was performed as described previously (1). Signal detection of protein bands was performed using LAS-4000 system (GE healthcare). The membrane after signal detection was subjected to Coomassie Brilliant Blue (CBB) staining to show loading control.

Quantitative Real-time PCR (qPCR)

Total RNA (200 ng) was isolated using TRIzol Reagent (Invitrogen) and subjected to reverse transcription with random hexamer and oligo dT primers using PrimeScript RT Reagent kit (Takara Bio, Japan). qPCR was performed in 20 µl aliquots of reaction mix using SYBR premix Ex Taq II (Takara Bio, Japan) and Thermal Cycler Dice Real Time System (Takara Bio, Japan). Relative gene expression values were calculated by delta-delta Ct method and normalized by the value obtained from 5S rRNA or *GAPDH* mRNA. Primer sequences used for qPCR are listed in Supplementary Table S2.

RNA interference (RNAi)

Stealth RNAi siRNA and scrambled-sequence RNA (scRNA; negative control) were purchased from Thermo Fisher Scientific, and Silencer Select siRNA and negative control RNA (ncRNA)

were from Ambion. Transfection of cells with the siRNAs was performed using Lipofectamine RNAiMax (Invitrogen). In 35-mm Petri dishes, 293T or T-REx 293 cells were transfected with 25 pmol of Silencer Select siRNA/ncRNA or 100 pmol of Stealth RNAi siRNA/scRNA, and HeLa or MCF7 cells with 12.5 pmol of Silencer Select siRNA/ncRNA or 50 pmol of Stealth RNAi siRNA/scRNA. All siRNA sequences are shown in Supplementary Table S2.

Chromatin immunoprecipitation (ChIP)

Cells were treated with 1% formaldehyde for 10 min at 37°C to cross-link proteins and DNA, and the reaction was quenched with 0.125 M glycine (final concentration) for 5 min. Cells were harvested and washed twice with phosphate-buffered saline (PBS). Nuclei, prepared via a three-step method as described (2), were suspended in buffer containing 50 mM Tris-HCl pH 8.0, 10 mM EDTA, 1% SDS, 1 mM phenylmethylsulfonyl fluoride, PMSF OR buffer D (50 mM Tris-HCl pH 8.0, 10 mM EDTA, 1 % SDS, 1 mM phenylmethylsulfonyl fluoride, PMSF) and sonicated to shear the chromatin using a Bioruptor 200 (CosmoBio, Japan; highest setting, 12 times for 30 s, 4°C). The sheared chromatin was collected by centrifugation at 20,000 × *g* for 10 min, incubated at 65°C for 12 h for decrosslinking, and purified with the QIAquick PCR Purification kit (Qiagen). DNA concentration in the chromatin solution was determined with an ND-1000 spectrophotometer (Nano-Drop Technologies, Wilmington, DE, USA). The solution containing 5 µg of sheared DNA was diluted 1:10 with ChIP dilution buffer (16.7 mM Tris-HCl pH 8.0, 0.01% SDS, 1.1% (w/v) Triton X-100, 1.2 mM EDTA, 167 mM NaCl, and 1 mM PMSF). ChIP was done with 3 µg of an appropriate antibody, with rotation overnight at 4°C. An aliquot (30 µl) of the diluted solution was used as an input control. Antibody was collected by binding to 25 µl of Protein G Dynabeads (Invitrogen) with incubation for 2 h at 4°C in the presence of 0.4 mg/ml bovine serum albumin and 0.25 mg/ml sheared salmon sperm DNA. The antibody-bound Dynabeads were washed using MagRack (GE Healthcare) twice with 1 ml low-salt wash buffer (20 mM Tris-HCl pH 8.0, 0.1% SDS, 1% Triton X-100, 2 mM EDTA, 150 mM NaCl), once with 1 ml high-salt wash buffer (20 mM Tris-HCl pH 8.0, 0.1% SDS, 1% Triton X-100, 2 mM EDTA, 400 mM NaCl), and twice with 1 ml TE buffer (10 mM Tris-HCl pH 8.0, 1 mM EDTA, 0.01% Triton X-100). The DNA-bound proteins were eluted by two successive incubations (15 min each, 37°C) with 0.1 M NaHCO3, 1% SDS, and 10 mM DTT. After adding NaCl to a final concentration of 200 mM, the DNA-bound protein was heated at 65°C for more than 6 h to reverse the formaldehyde crosslinks. The protein sample was digested with proteinase K (final concentration 50 µg/ml) for 1 h at 50°C. The released DNA was purified using the QIAquick PCR Purification kit, and target DNA fragments were amplified by real-time PCR using a thermal cycler (Dice TP800) and SYBR Premix Ex Taq II (TAKARA Bio Inc., Japan). The primer sets used for ChIP were listed in Supplementary Table S2. For re-ChIP analysis, the first immunoprecipitate with anti-LYAR-FLAG was collected with 12 µl of FLAG M2 magnetic beads (Sigma) and eluted with FLAG peptide as described in the supplementary materials. After diluting the eluate (100 µl) with 900 µl ChIP dilution buffer, an aliquot of 450 µl was subjected to

a second ChIP with anti-BRD2 or rabbit IgG (control). The subsequent steps were as described above.

Construction of epitope-tagged expression vectors

Construction of HBF-LYAR-pcDNA5FRT/TO, DNA fragment encoding LYAR was amplified by KOD-Plus-neo (Toyobo, Japan) with the primer set (LYAR_1-379-for/-rev) using FLAG-LYAR-pcDNA3.1(+)(3) as a template DNA. The amplified DNA was digested with BamHI and XhoI, and inserted into BamHI / XhoI site of DAP-TDP-43 pcDNA5/FRT/TO (4). For the construction of HBF-pcDNA5FRT/TO, DNA fragment containing TGA sequence, encoding stop codon, was generated by annealing with the primer sets (TGA-for/-rev), and inserted into BamHI / XhoI site of DAP-TDP-43 pcDNA5/FRT/TO (4). For the construction of each of HBF-tagged LYAR deletion mutants (LYAR1-167, LYAR168-379, LYAR168-260, or LYAR261-379), by using HBF-LYAR-pcDNA5/FRT/TO as a template DNA, each of DNA fragments encoding deletion mutants was amplified by KOD-Plus-Neo using a primer sets (LYAR_1-167-for/-rev, LYAR_168-379-for/-rev, LYAR_168-260-for/-rev or LYAR_261-379-for/-rev). The amplified DNA was digested with BamHI and XhoI, and inserted into BamHI / XhoI site of DAP-TDP-43 pcDNA5/FRT/TO. pCold-TF DNA (Clontech) was used to prepare His6-Triger factor (TF)–fused recombinant proteins. pCold-TF-FLAG vector encoding TF-FLAG or pCold-TF-TEV-HA vector encoding TF-HA was constructed by a method described previously (5). For the construction of pClod-TF-LYAR-FL, DNA fragment encoding full length LYAR was amplified by KOD-Plus-Neo with the primer set (TF-LYAR-for/-rev) and HBF-LYAR pcDNA/FRT/TO as a template. The amplified DNA was digested with NdeI and BamHI, and inserted into NdeI / BamHI site of pCold-TF-FLAG vector. For the construction of pClod-TF-LYAR$_{168-260}$-HA, DNA fragment encoding LYAR$_{168-260}$ was amplified using KOD-Plus-Neo with the primer set (TF-LYAR_168-260-for/-rev) and HBF-LYAR pcDNA/FRT/TO as a template. The amplified DNA was digested with BamHI and HindIII, and inserted into BamHI / HindIII site of pCold-TF-TEV-HA vector. To prepare BRD2 recombinant protein, DNA fragment encoding BRD2 was amplified by KOD-Plus-Neo with the primer set (BRD2-for/-rev) and cDNA library derived from 293T cells as a template DNA. The amplified DNA was digested with KpnI and XbaI, and inserted into KpnI / XbaI site of pBIND (Promega). The resulting vector was named “pBIND-BRD2”. For the construction of pCold-TF vectors encoding TF-fused BRD2 and its deletion mutants (BRD2$_{1-801}$, BRD2$_{1-457}$, BRD2$_{458-801}$, BRD2$_{458-720}$, BRD2$_{458-600}$, or BRD2$_{601-801}$), DNA fragment encoding a mutant fused to FLAG tag at the C-terminal was amplified using KOD-Plus-Neo with the primer set corresponded to BRD2_1-801-for/-rev, BRD2_1-457-for/-rev, BRD2_458-801-for/-rev, BRD2_458-720-for/-rev, or BRD2_458-600-for/-rev, and pBIND-BRD2 as a template DNA. The amplified DNA was digested with KpnI and XbaI, and inserted into KpnI / XbaI site of pCold-TF vector. The resulting vector encoding BRD2$_{1-801}$ was named “pCold-TF-BRD2-FLAG”. For the construction of vector encoding BRD2$_{601-720}$, DNA fragment encoding BRD2$_{601-720}$ fused to FLAG tag at the C-terminal was amplified using KOD-Plus-Neo with the primer set (BRD2_602-720-for/-rev) and pBIND-BRD2 as a template DNA. The amplified DNA was digested with KpnI and XbaI, and

inserted into KpnI / XbaI site of pCold-I vector (Clonthch). For the construction of HBF-BRD2 pcDNA5/FRT/TO, DNA fragment encoding BRD2-FLAG was digested with KpnI and XhoI form pCold-TF-BRD2-FLAG, and inserted into KpnI / XhoI site of HBF-LYAR-pcDNA5FRT/TO. For the construction of FLAG-BRD4-pcDNA5/FRT/TO, BRD4 DNA fragment was amplified using KOD-Plus-Neo with the primer set (BRD4-for/-rev) and cDNA from 293T cells as a template DNA. The amplified DNA was digested with BamHI and XhoI, and inserted into BamHI / XhoI site of FLAG-Chtop-pcDNA5/FRT/TO (1). The primer sets used for construction of expression vectors were listed in Supplementary Table S2. The sequence of constructed vectors was confirmed by DNA sequencing.

Construction of doxycycline-inducible cell lines

T-REx 293 cell lines expressing doxycycline inducible protein were established as described previously (1). To induce expression of the protein, 100 ng/ml of doxycycline was treated to the cells for the indicated time periods.

Immunocytostaining

Immunocytostaining was performed based on the method as described previously (4). Briefly, cells were grown on collagen-coated culture slides. After removal of the culture medium, the cells were washed with PBS and fixed with 4 % paraformaldehyde in PBS for 10 min at 25 °C. After washing twice with PBS containing 0.05% (w/v) Tween 20 (PBST), the cells were permeabilized with PBS containing 0.1% (v/v) Triton X-100 (SIGMA) for 5 min at 25 °C, and then washed again with PBST. The cells were blocked with 3 % (w/v) non-fat dried skim milk in PBST for 1 h, and then incubated with the appropriate primary antibody for 2 h at 25 °C. After washed three times with PBST for 10 min, the cells were incubated with a fluorescence-conjugated secondary antibody for 1 h at 25 °C. Finally, after washing three times with PBST for 10 min, the cells were mounted using VECTASHIELD Mounting Medium with DAPI (Vector Laboratories, Burlingame, CA), and examined with an Axiovert 200 M microscope (Carl Zeiss, Oberkochen, Germany).

HpaII resistant assay

The purified input DNA obtained from Chromatin immunoprecipitation assay was used for HpaII resistant assay. DNA was treated with HpaII enzyme (Takara Bio, Japan) for 2 h at 37 °C according to the manufacturer's instructions. As a control experiment, equal aliquot of input DNA was treated without HpaII enzyme. The resulting DNA was purified using QIAquick PCR Purification kit (Qiagen) and H0 or H8 rDNA fragments were amplified by quantitative real-time PCR using a Thermal Cycler Dice Real Time System and SYBR Premix Ex Taq II (Takara Bio, Japan). The primer sets to detect H0 and H8 fragments were listed in Supplementary Table S5.

prHu3-Luciferase assay

The luciferase reporter plasmid, prHu3-Luc, was a gift from Dr. Yan-Hwa Wu Lee (National Yang-Ming University, Taipei, Taiwan) (6). pRL-TK (Promega) was used as an internal reporter

gene. 293T cells at 60% confluency that had been cultured in a 6-well plate were co-transfected with 250 ng of prHu3-Luc, 250 ng of pRL-TK, and the indicated amount of HBF-LYAR pcDNA5FRT/TO (HBF, His6-biotinylation sequence-FLAG tag) and/or HBF pcDNA5FRT/TO using 4 µl of Lipofectamine 2000 (Invitrogen). After 24 h of transfection, cells were collected and subjected to luciferase assay using the Dual-Glo Luciferase Assay System (Promega) and the GloMax Multi Detection System (Promega).

Sucrose Density Gradient Ultracentrifugation

Sucrose density gradient ultracentrifugation was performed as described previously (7).

Cellular fractionation

Cells at 80% confluency were collected with ice-cold PBS and lysed with a buffer (16.7 mM Tris-HCl pH 7.4, 50 mM NaCl, 1.67 mM $MgCl_2$, 1 mM PMSF, 2 µg/ml aprotinin, 2 µg/ml pepstatin A and 2 µg/ml leupeptin) containing 0.1% Triton X-100 for 5 min on ice. After centrifugation at 1,000 × *g* for 5 min, the supernatant was collected as the cytoplasmic extract. The pellet was washed once with the same buffer, and resuspended with a buffer containing 50 mM Tris-HCl pH 7.4, 150 mM NaCl, 1 mM PMSF, 2 µg/ml aprotinin, 2 µg/ml pepstatin A, 2 µg/ml leupeptin, and 0.5% w/v IGEPAL CA-630. The pellet-resuspended solution was adjusted to 400 mM NaCl by adding 2.5 M NaCl, incubated for 10 min on ice, and centrifuged at 20,000 × *g* for 10 min at 4°C to collect the supernatant as the nuclear extract. To prepare total cell extract, the collected cells were lysed with SDS sample buffer, sonicated using the Bioruptor 200 (highest setting, five times for 30 s at 4°C), and centrifuged at 20,000 × *g* for 10 min. The supernatant was collected as the total cell extract.

Purification of HBF-LYAR complexes

After 24 h doxycycline treatment, 5 x 15 mm dish of T-REx 293 cells expressing HBF-LYAR (or untransfected T-REx 293 cells as a control) were harvested and washed twice with ice cold PBS. The harvested cells were subjected to cellular fractionation, and nuclear extract was prepared as described in Cellular fractionation. The nuclear extract (10 mg) was incubated with 50 µl of Ni-NTA agarose (Qiagen) for 1 h at 4 °C. After Ni-NTA Agarose was washed five times with wash buffer (50 mM Tris-HCl pH 8.0, 150 mM NaCl, 0.5% IGEPAL CA-630), Ni-NTA Agarose was treated with 100 µg/ml RNase A in a solution containing 50 mM Tris-HCl pH 8.0 and 150 mM NaCl for 30 min at 37°C. After washing three times with wash buffer, HBF-LYAR complexes were eluted twice from the Ni-NTA Agarose with 100 µl of elution buffer (50 mM Tris-HCl pH 8.0, 150 mM NaCl, 250 mM imidazole) on ice for 10 min. The eluted solution, that was diluted with 400 µl of wash buffer containing 1 mM PMSF, was incubated with 20 µl Anti-FLAG M2 Affinity Gel (Sigma-Aldrich) for 4 h at 4 °C. After washing the gel five times with wash buffer, the HBF-LYAR complexes were eluted twice from the gel with 75 µl of elution buffer (500 µg/ml FLAG peptide, 50 mM Tris-HCl pH 8.0 and 150 mM NaCl). One-third of the

HBF-LYAR complex eluate was subjected to SDS-PAGE and subsequent silver staining. The remaining two-third of the HBF-LYAR complex eluate was analyzed by LC-MS/MS.

LC-MS/MS Analysis

The purified LYAR-associated complexes were separated by SDS-PAGE using 7.5 % - 20 % gradient gel, and stained with Colloidal Blue Staining Kit (Invitrogen). The gel was cut into four pieces, and each of the gel pieces was reduced with DTT, alkylated with iodoacetamide, and subjected to in-gel digestion with Trypsin/Lys-C Mix Mass Spec Grade (Promega). The resulting peptides were analyzed by a nanoscale LC-MS/MS system using LTQ-Orbitrap hybrid MS (model XL, Thermo Fisher Scientific) as described (7,8). Raw data were acquired using Xcalibur v2.0.7 (Thermo Fisher Scientific) and converted to a Mascot generic format (mgf) file by Proteome Discoverer v1.3 (Thermo Fisher Scientific). The protein prediction search was performed by Mascot v2.4.0 (www.matrixscience.com) against the UniProt human database SwissProt_2014_02 and using the following parameters: fixed modification, carbamidomethyl (C); variable modifications, oxidation (M), N-terminal acetylation (Acetyl N-term), Gln->pyro-Glu (N-term Q); allow up to three missed cleavage; peptide mass tolerance, 10 ppm; MS/MS tolerance, 0.8 Da. We selected candidate peptides on the basis of their probability-based Significance threshold ($p < 0.05$); these were referred to as “hits”. Criteria for selection were based on definitions provided in Mascot. In addition, more stringent criteria were applied for protein assignment, as follows: (i) any protein with < 4 peptide candidates was eliminated from a hit candidate; (ii) peptide candidates that were common between a group of proteins were excluded from the hit candidates. The Proteins identified from the control sample (Flp-In T-RE-x 293 cells) were not included in the HBF-LYAR-associated proteins, unless > 4 fold increase of prot_matches_sig (Count of PSMs that have significant scores) was confirmed. False discovery rate of this analysis was 0.0177 (Number of matches above identity threshold in search of real database was 2536, whereas number of matches above identity threshold in search of decoy database was 45).

Northern blot analysis

Northern blot analysis was performed as described previously (1). Biotin labeled DNA probe (5’- TTCCGAGATCAGACGAGATCGG -3’) was used for the detection of 5S rRNA.

Immunoprecipitation with His6-FLAG tagged protein

Nuclear extract, prepared as described in Cellular fractionation of the main text, was incubated with 30 µl of Ni-NTA agarose (Qiagen), and washed once with wash buffer (50 mM Tris-HCl pH 8.0, 150 mM NaCl, 0.5% IGEPAL CA-630), for 1 h at 4 °C. After Ni-NTA Agarose was washed five times with wash buffer, His6-tagged protein complexes were eluted twice from the Ni-NTA Agarose with 100 µl of elution buffer (50 mM Tris-HCl pH 8.0, 150 mM NaCl, 250 mM imidazole) on ice for 10 min. The eluate was diluted with 400 µl of wash buffer containing 1 mM PMSF, and was incubated with 15 µl FLAG M2 Magnetic beads (Sigma-Aldrich) for 4 h at 4 °C.

After washing the beads five times with wash buffer, the FLAG-tagged protein complexes were eluted with SDS sample buffer for 3 min at 95 ºC and subjected to the immunoblot analysis. For immunoprecipitation of FLAG-BRD4 complexes, internal His6 sequence of BRD4 (738-743 a.a. residues) was used to pull down with Ni-NTA agarose for the first step purification.

Immunoprecipitation with anti-UBF or anti-SPT5 antibody

HBF-LYAR complexes were two-step purified using Ni-NTA agarose and FLAG M2 beads as above except for the elution with 100 µl of elution buffer (500 µg/ml FLAG peptide, 50 mM Tris-HCl pH 8.0 and 150 mM NaCl). For the immunoprecipitation of endogenous UBF or SPT5, 3 µg of anti-UBF antibody or nonspecific mouse IgG, or anti-SPT5 antibody or nonspecific rabbit IgG as a control was incubated with 15 µl of Protein G Dynabeads (Thermo Fisher Scientific) in PBST at RT for 30 min. After washing the beads three times with PBST, antibody-bound Dynabeads were subjected to the immunoprecipition of UBF or SPT5. HBF-LYAR complex eluate was diluted with 400 µl of wash buffer containing 1 mM PMSF, and incubated with anti-UBF or anti-SPT5 antibody –bound Dynabeads for 4 h at 4 °C. After washing the beads five times with wash buffer, UBF or SPT5 complexes were eluted with SDS sample buffer for 3 min at 95 ºC and subjected to the immunoblot analysis.

Preparation of recombinant proteins

Triggering factor (TF)-fused proteins were expressed in *Escherichia coli* Rosetta2 (DE3) pLysS cell lines (Novagen) via a 24-h induction at 15 °C in the presence of 0.1 mM isopropyl-β-D-1-thiogalactosylparanoside. Cells were extracted with xTractor Buffer (Clontech), and TF-fused proteins were pulled down using Ni-NTA agarose (Qiagen) and the TALON Magnetic Beads Buffer kit (Clontech). For further purification, the TF-fused proteins, which were C-terminally tagged with either FLAG or HA, were subjected to immunoprecipitation with Anti-FLAG M2 Affinity Gel (SIGMA) or monoclonal Anti-HA-Agarose (Sigma Aldrich). After washing five times with wash buffer (50 mM sodium phosphate pH 7.2, 300 mM NaCl, 0.01 % Triton X-100), FLAG- and HA-tagged proteins were eluted with 500 µg/ml of FLAG peptide and 200 µg/ml of HA peptide, respectively, in the solution containing 50 mM sodium phosphate pH 7.2, and 300 mM NaCl. Each eluate was equilibrated to the solution (50 mM Tris-HCl pH 7.4 and 150 mM NaCl) using Zeba Spin Desalting columns (7 kDa molecular weight cutoff, Thermo Scientific) and used for *in vitro* binding assays.

Electrophoretic mobility shift assay (EMSA)

Biotin-labeled DNA probe was amplified using Ex Taq II (TAKARA Bio, Japan), biotin-labeled primers and Input DNA obtained by ChIP analysis of 293T cells as a template DNA. The amplified DNA was purified using MinElute PCR Purification Kit (Qiagen), and the DNA concentration was determined by ND-1000 spectrophotometer (Nano-Drop Technologies, Wilmington, DE, USA). Biotin-labeled primers were listed in Supplementary Table S5. EMSA was performed using LightShift EMSA Optimization and Control kit (Thermo Fisher Scientific). Biotin-labeled DNA

probe (20 fmol) and recombinant protein (500 ng) were incubated for 30 min at room temperature in 20 μl of reaction buffer containing 10 mM Tris-HCl (pH 7.4), 50 mM KCl, 1 mM DTT, 2.5 % Glycerol, 0.05 % NP-40, and 50 ng/μl poly(dI·dC). The reactiants were run on 5% native polyacrylamide gel in 0.5 x TBE buffer (Tris/Borate/ EDTA), and transferred to a Hybond N+ membrane (GE Healthcare). The membrane was dried, and cross-linked using an ultraviolet cross linker (CX-2000, UVC, CA, USA) at 120 mJ/cm^2. Biotin-labeled DNA probe was detected with Streptavidin-HRP Conjugate and Chemiluminescent Nucleic Acids Detection Module Kit (Thermo Fisher Scientific, 89880D). Signals were detected with an LAS-4000 system.

In vitro binding assay

Hemagglutinin (HA)-tagged and FLAG-tagged recombinant proteins (400 ng each) were mixed in 20 μl binding buffer containing 50 mM Tris-HCl pH 7.4, 150 mM NaCl, and 0.01% (v/v) Triton X-100 for 30 min at 25°C. After adding 30 μl more of binding buffer, the recombinant protein mixture was incubated with shaking in 15 μl of anti-HA–coupled agarose (Sigma Aldrich) solution for 1 h at 25°C. After washing four times with binding buffer, HA-tagged recombinant protein was eluted with 200 μg/ml of HA peptide in 50 mM Tris-HCl pH 7.4 and 150 mM NaCl. The eluted proteins were subjected to SDS-PAGE and to immunoblotting with anti-HA or anti-FLAG.

Protein expression and purification for ITC binding assay

The genes of human BRD2 and LYAR were cloned into a modified pET28-MKH8SUMO vector to express BRD2 and LYAR with a N-terminal Hexa-His and Sumo-tag and a TEV cleavage site. The recombinant BRD2 and LYAR constructs were transformed into an E. coli BL21(DE3) codon plus RIL strain, and the cells were cultured with Terrific broth medium and induced with 0.2 mM IPTG at 16 °C overnight. BRD2 and LYAR were respectively purified by Ni^{2+}-NTA affinity, ion exchange and Superdex 200 10/300 chromatography (GE Healthcare). The buffer for gel filtration contained 20 mM Tris-HCl (pH 7.36), 150 mM NaCl, and 1 mM DTT. The peak fractions of BRD2 and LYAR were respectively collected and concentrated to about 20 mg/ml and stored at -80 °C for further use.

ITC binding assay

ITC measurements were performed with VP-ITC microcalorimeter (MicroCal) at 25 °C. The purified BRD2 were diluted to 50 μM using ITC buffer (20 mM Tris-HCl pH 7.36, 150 mM NaCl, and 1 mM DTT) and loaded into the cell chamber. The purified LYAR were diluted to 1 mM using ITC buffer, and twenty-five injections of 10 μl LYAR were carried out with a spacing of 180 sec and a reference power of 15 μcal/sec. OriginLab Pro7 software was employed for data analysis.

SUPPLEMENTARY FIGURE LEGENDS

Supplementary Figure S1.

A) The GeneChip score for LYAR mRNA expression is given for the indicated normal and tumor tissues. HCC, hepatocellular carcinoma; SCLC, small-cell lung carcinoma; SqLC, squamous cell lung carcinoma; AdLC, adenocarcinoma lung cancer; RCC, renal cell carcinoma; CRC, colorectal carcinoma; Panc Ca, pancreatic cancer. B) The level of LYAR mRNA, as assessed with reverse transcription-PCR, was compared between normal (N) and tumor (T) primary foci obtained from each of four different patients (CRC01, CRC02, CRC03, CRC04) who had colon cancer. The GeneChip score and the relative increase in LYAR mRNA level (T/N) are given for each tissue. C) Immunoblotting (left) and reverse transcription-PCR (right) of LYAR and BRD2 levels in HeLa, SW480, and SW620 cells. Each whole-cell extract was subjected to immunoblotting with antibodies indicated to the right of the panels. Coomassie brilliant blue staining of the membrane was used to assess equal loading of lanes. The graph shows the level of each mRNA (indicated under the bars) relative to that measured for HeLa cells. Each mRNA level was normalized to that of 5S rRNA. Data represent the mean ± SEM of three independent experiments. $^{**}P < 0.01$ (unpaired t-test).

Supplementary Figure S2.

A, B) ChIP analysis of LYAR binding to rDNA loci in MCF7 and 293T cells. MCF7 (A) or 293T cells (B) were subjected to ChIP analysis with anti-LYAR or nonspecific rabbit IgG (control). The data represent the amount of ChIPed DNA (% of input) for the rDNA regions indicated under the graph. Data represent the mean ± SEM of three independent experiments. C) MCF7 cells were treated with an siRNA specific for LYAR and then subjected to ChIP analysis with anti-LYAR or anti-UBF. LYAR knockdown was examined by immunoblotting. Lamin B was used as the loading control. The graphs show the amount of ChIPed DNA (% of input). Data represent the mean ± SEM of three independent experiments. D) Immunoblotting for HBF-LYAR in HBF-LYAR-TO cells. HBF-LYAR-TO cells were treated with Dox for the indicated periods, and HBF-LYAR levels were confirmed by immunoblotting (IB) with anti-LYAR. UBF and GAPDH were used as loading controls. The graph shows band intensities for HBF-LYAR relative to endogenous LYAR. E) Immunocytostaining for HBF-LYAR and UBF upon HBF-LYAR overexpression. HBF-LYAR-TO cells were treated with Dox for the indicated periods and then subjected to immunocytostaining with anti-FLAG (rabbit IgG, for LYAR, green) and anti-UBF (mouse IgG, red). FITC-conjugated anti-rabbit IgG and Cy3-conjugated anti-mouse IgG were used as the secondary antibodies. T-REx 293 cells treated with Dox were used as the control. DAPI was used to stain the nucleus. Bar: 5 µm.

Supplementary Figure S3.

A) Nuclear extract (1 mg) prepared from Dox-treated HBF-LYAR-TO cells was separated into 20 fractions by ultracentrifugation with a 10–30% sucrose density gradient. Absorbance was

monitored at 260 nm. Equivalent amounts of proteins in each fraction were analyzed by immunoblotting (IB) with the antibodies indicated to the right. B) Cellular fractionation of HBF-LYAR-TO cells. HBF-LYAR-TO cells or T-REx 293 cells (control) were treated with Dox for 24 h and then subjected to cellular fractionation. Total, whole-cell extract; Cyt, cytoplasmic fraction; Nuc, nuclear fraction. The localization of HBF-LYAR was confirmed by immunoblotting with antibodies indicated to the right. HBF-LYAR was detected using Stabilized Streptavidin-HRP conjugate. GAPDH and lamin B were used as cytoplasmic and nuclear markers, respectively. Molecular mass markers (kDa) are indicated to the left. C) HBF-LYAR-associated complexes for liquid chromatography–coupled MS/MS analysis were confirmed to be RNA-free complexes by immunoblotting or northern blotting. Protein and RNA components from HBF-LYAR-associated complexes with or without RNase A treatment were extracted and subjected to immunoblotting with anti-nucleolin, to the Stabilized Streptavidin-HRP Conjugate (to detect HBF-LYAR), or to northern blotting using a 5S rRNA probe (lower panel). D) The Gene Ontology terms (Biological process and Molecular function) of HBF-LYAR-associated proteins identified by liquid chromatography–coupled MS/MS analysis using DAVID.

Supplementary Figure S4.

A) Electrophoretic mobility shift assay to detect binding between LYAR and rDNA fragments. TF (trigger factor)-LYAR-FL (FLAG) or TF-FL (control) was incubated with biotin-labeled H0 or H8 probe and then subjected to native polyacrylamide gel electrophoresis. The biotin-labeled probes were detected with chemiluminescence. B) Schematic diagram of the LYAR domain mutants, and immunoblotting of proteins that bind these mutants. Amino acid residue numbers are shown at the top. Each of the LYAR domain mutant cells (HBF-LYAR-TO cells) was treated with Dox for 24 h, and proteins associated with HBF-LYAR domain mutants were subjected to two-step pulldown using His6 and FLAG-tag and RNase A treatment. Dox-treated T-REx 293 cells served as the control. Proteins that could bind the HBF-LYAR domain mutants were detected by immunoblotting with antibodies indicated to the right of the panels. HBF-tagged proteins were detected with Stabilized Streptavidin-HRP Conjugate. Molecular mass markers are shown at the left. C) ChIP analysis of LYAR168–379 binding to rDNA loci. HBF-LYAR168–379-TO cells were treated with or without Dox for 24 h and then subjected to ChIP analysis with anti-FLAG. The data reflect the amount of ChIPed DNA (% of input), as indicated. Data represent the mean ± SEM of three independent experiments. *$P < 0.05$, **$P < 0.01$ (unpaired t-test). D) ChIP analysis of LYAR binding to rDNA loci upon treacle knockdown. 293T cells were treated with a treacle-specific siRNA for 72 h and then subjected to ChIP analysis with anti-LYAR. Treacle or LYAR levels upon treacle knockdown were confirmed by immunoblotting with anti-treacle or anti-LYAR. GAPDH was used as the loading control. The data reflect the relative amount of ChIPed DNA, as indicated. Data represent the mean ± SEM of three independent experiments. **$P < 0.01$ (unpaired t-test). E) Metabolic labeling (4-thiouridine) of newly synthesized 47/45S pre-rRNA in 293T cells treated with 100 ng/ml

actinomycin D (actD) or 2 μM CX-5461 for 2 h. The pre-rRNA was biotinylated and then subjected to agarose gel electrophoresis under denaturing condition and northern blotting. Signals for 47/45S pre-rRNA were detected by chemiluminescence. 28S and 18S rRNAs were used as loading controls (stained with methylene blue). F) The nuclear extracts from the dox–treated HBF-LYAR-TO cells with or without 100 ng/ml actD or 2 μM CX-5461 for 2 h or 6 h were subjected to two-step pulldown with His6-tag and FLAG-tag of HBF-LYAR and RNase A treatment. HBF-LYAR-associated proteins were confirmed with the antibodies indicated to the right. HBF-LYAR was detected using Stabilized Streptavidin-HRP Conjugate. G) ChIP analysis of LYAR binding to rDNA loci. 293T cells were treated with or without 100 ng/ml actD for 2 h and then subjected to ChIP analysis with anti-LYAR. The data reflect the amount of ChIPed DNA (% of input), as indicated. Data represent the mean ± SEM of three independent experiments. *P < 0.05 (unpaired t-test).

Supplementary Figure S5.

A) ChIP analysis of the binding of LYAR-associated proteins to rDNA loci upon LYAR knockdown (the left-most five graphs). HeLa cells were treated with a LYAR-specific siRNA for 96 h and then subjected to ChIP with antibodies indicated under the graphs. ChIP analysis of SPT5 binding to rDNA loci upon the overexpression of LYAR is shown at the right. The data reflect the amount of ChIPed DNA, as indicated. Data represent the mean ± SEM of at least three independent experiments. B) ChIP analysis of BRD2 binding to rDNA loci upon knockdown of BRD2. 293T cells were treated with an siRNA specific for BRD2 for 72 h and then subjected to ChIP analysis with anti-BRD2. The levels of BRD2, BRD4, and LYAR were confirmed by immunoblotting with the indicated antibodies. β-actin was used as the loading control. The data reflect the amount of ChIPed DNA (% of input), as indicated. Data represent the mean ± SEM of three independent experiments. *P < 0.05, **P < 0.01 (unpaired t-test). C) Schematic representation of BRD2 domain mutants used in the in vitro binding assay and isothermal titration calorimetry binding assay (left panel). TF-tagged recombinant proteins, purified via use of His6-tag, HA-tag, or FLAG-tag, were visualized by colloidal blue staining (right panel). D) In vitro binding assay to assess the binding between LYAR168–260 and BRD2 domain mutants. The assay was performed by incubating TF-LYAR168–260-HA with TF-BRD2-FL mutants, followed by IP with anti-HA. TF-BRD2-FLAG mutants were detected by immunoblotting with anti-FLAG. As a control for TF-LYAR168-260-HA, TF-HA was used, and for TF-BRD2-FL, TF-FL was used. E) Isothermal titration calorimetry binding assay using purified LYAR (LYAR175–219 or LYAR168–210) and BRD2 (BRD2489–540). Buffer alone was titrated into BRD2 as a negative control. F) ChIP analysis of BRD2 (left) or BRD4 (right) binding to rDNA loci in HBF-LYAR-TO cells. HBF-LYAR-TO cells were treated with or without Dox for 24 h and then subjected to ChIP analysis with anti-BRD2 or anti-BRD4. The levels of BRD2, BRD4, and HBF-LYAR were confirmed by immunoblotting with the indicated antibodies. β -actin was used as the loading control. The data reflect the amount of ChIPed DNA, as indicated. Data represent the mean ± SEM of three independent experiments. *P < 0.05, **P < 0.01

(unpaired t-test). G) ChIP analysis of LYAR (left) or UBF (right) binding to rDNA loci in HBF-BRD2-TO cells. HBF-BRD2-TO cells were treated with or without Dox for 24 h and then subjected to ChIP analysis with anti-LYAR or anti-UBF. The levels of LYAR, UBF, and HBF-BRD2 were confirmed by immunoblotting. β-actin was used as the loading control. The data reflect the amount of ChIPed DNA, as indicated. Data represent the mean ± SEM of three independent experiments. *P < 0.05, **P < 0.01 (unpaired t-test).

Supplementary Figure S6.

A) ChIP analysis of BRD4 binding to rDNA loci upon knockdown of BRD4. 293T cells were treated with an siRNA specific for BRD4 for 72 h and then subjected to ChIP analysis with anti-BRD4. The levels of BRD2, BRD4, and LYAR were confirmed by immunoblotting. β-actin was used as the loading control. The data reflect the relative amount of ChIPed DNA (% of input), as indicated. Data represent the mean ± SEM of three independent experiments. **P < 0.01 (unpaired t-test). B) ChIP analysis of GCN5 (left) or p300 (right) binding to rDNA loci in HBF-LYAR-TO cells. HBF-LYAR-TO cells were treated with or without Dox for 24 h and then subjected to ChIP analysis with anti-GCN5 or anti-p300. The data reflect the amount of ChIPed DNA (% of input), as indicated. Data represent the mean ± SEM of three independent experiments.

SUPPLEMENTARY REFERENCE

1. Izumikawa, K., Yoshikawa, H., Ishikawa, H., Nobe, Y., Yamauchi, Y., Philipsen, S., Simpson, R.J., Isobe, T. and Takahashi, N. (2016) Chtop (Chromatin target of Prmt1) auto-regulates its expression level via intron retention and nonsense-mediated decay of its own mRNA. *Nucleic Acids Res*, **44**, 9847-9859.
2. Bierhoff, H., Dammert, M.A., Brocks, D., Dambacher, S., Schotta, G. and Grummt, I. (2014) Quiescence-induced LncRNAs trigger H4K20 trimethylation and transcriptional silencing. *Mol Cell*, **54**, 675-682.
3. Miyazawa, N., Yoshikawa, H., Magae, S., Ishikawa, H., Izumikawa, K., Terukina, G., Suzuki, A., Nakamura-Fujiyama, S., Miura, Y., Hayano, T. *et al.* (2014) Human cell growth regulator Ly-1 antibody reactive homologue accelerates processing of preribosomal RNA. *Genes Cells*, **19**, 273-286.
4. Izumikawa, K., Nobe, Y., Yoshikawa, H., Ishikawa, H., Miura, Y., Nakayama, H., Nonaka, T., Hasegawa, M., Egawa, N., Inoue, H. *et al.* (2017) TDP-43 stabilises the processing intermediates of mitochondrial transcripts. *Sci Rep*, **7**, 7709.
5. Sato, S., Ishikawa, H., Yoshikawa, H., Izumikawa, K., Simpson, R.J. and Takahashi, N. (2015) Collaborator of alternative reading frame protein (CARF) regulates early processing of pre-ribosomal RNA by retaining XRN2 (5'-3' exoribonuclease) in the nucleoplasm. *Nucleic Acids Res*, **43**, 10397-10410.
6. Kao, C.F., Chen, S.Y., Chen, J.Y. and Wu Lee, Y.H. (2004) Modulation of p53 transcription regulatory activity and post-translational modification by hepatitis C virus core protein. *Oncogene*, **23**, 2472-2483.
7. Yoshikawa, H., Komatsu, W., Hayano, T., Miura, Y., Homma, K., Izumikawa, K., Ishikawa, H., Miyazawa, N., Tachikawa, H., Yamauchi, Y. *et al.* (2011) Splicing factor 2-associated protein p32 participates in ribosome biogenesis by regulating the binding of Nop52 and

fibrillarin to preribosome particles. *Mol Cell Proteomics*, **10**, M110 006148.
8. Izumikawa, K., Ishikawa, H., Yoshikawa, H., Terukina, G., Miyazawa, N., Nakayama, H., Nobe, Y., Taoka, M., Yamauchi, Y., Philipsen, S. *et al.* (2014) Friend of Prmt1, FOP is a Novel Component of the Nuclear SMN Complex Isolated Using Biotin Affinity Purification. *Journal of Proteomics & Bioinformatics*, **07**.

Supplementary Figure S1

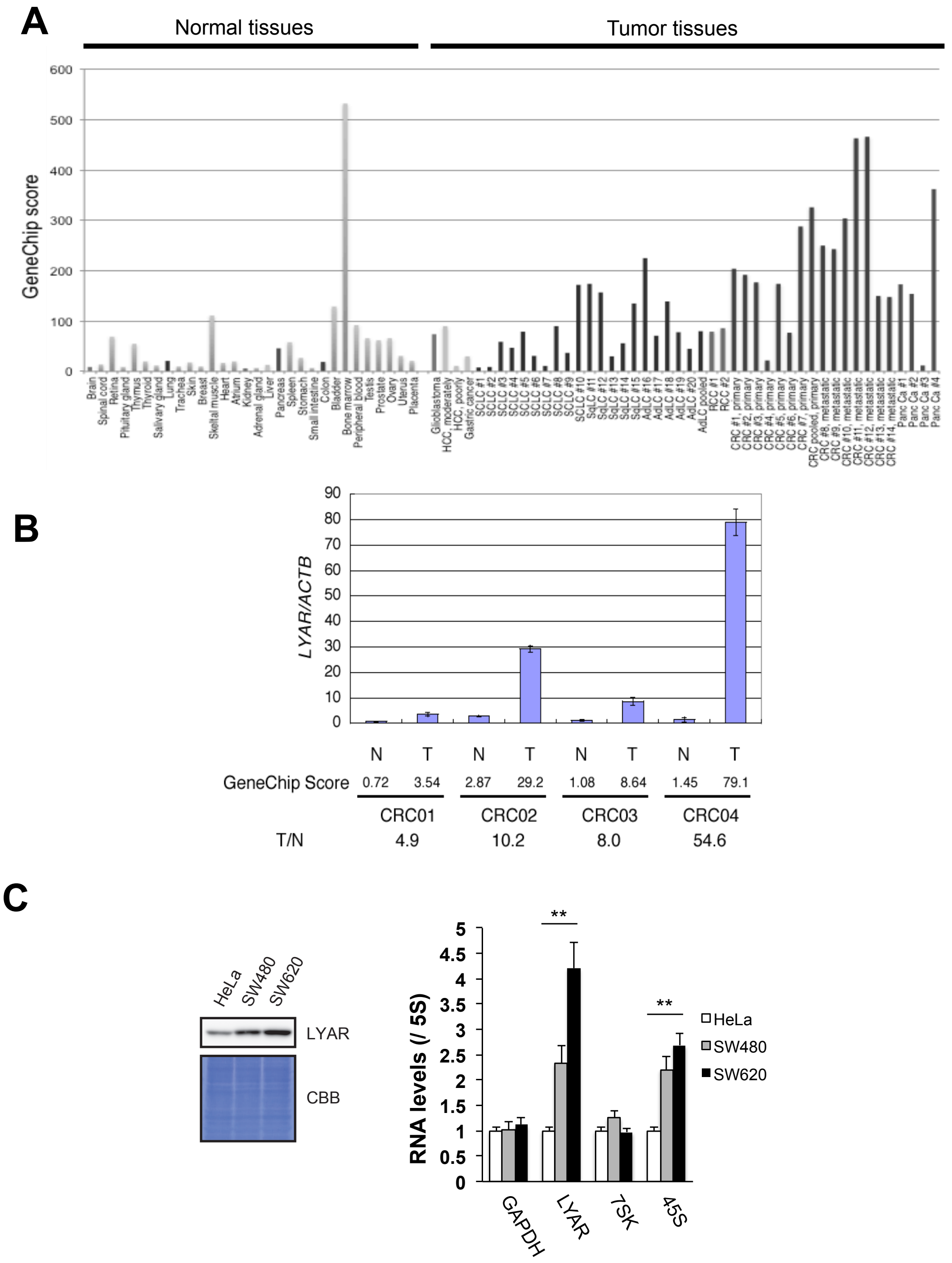

Supplementary Figure S2

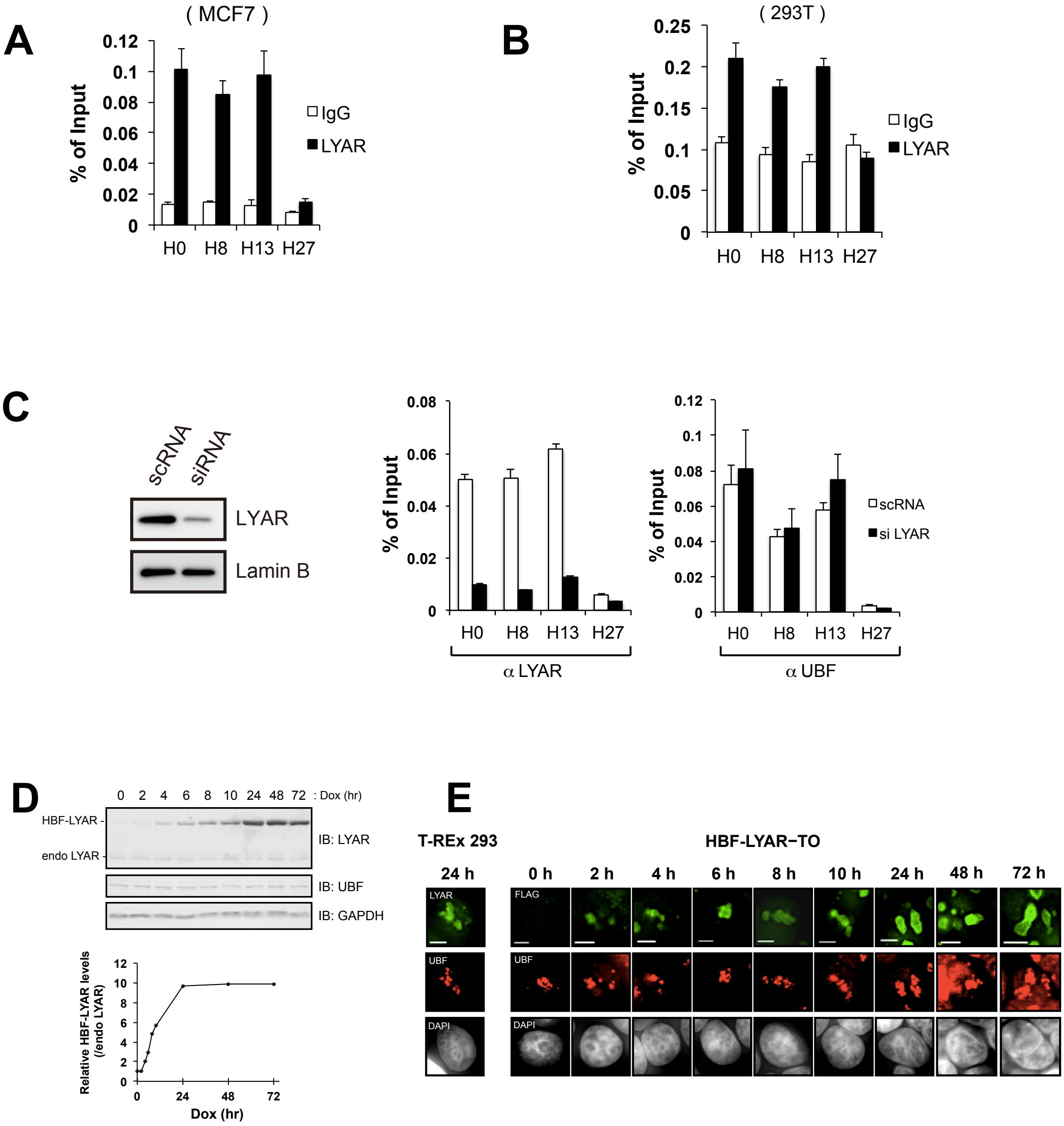

Supplementary Figure S3

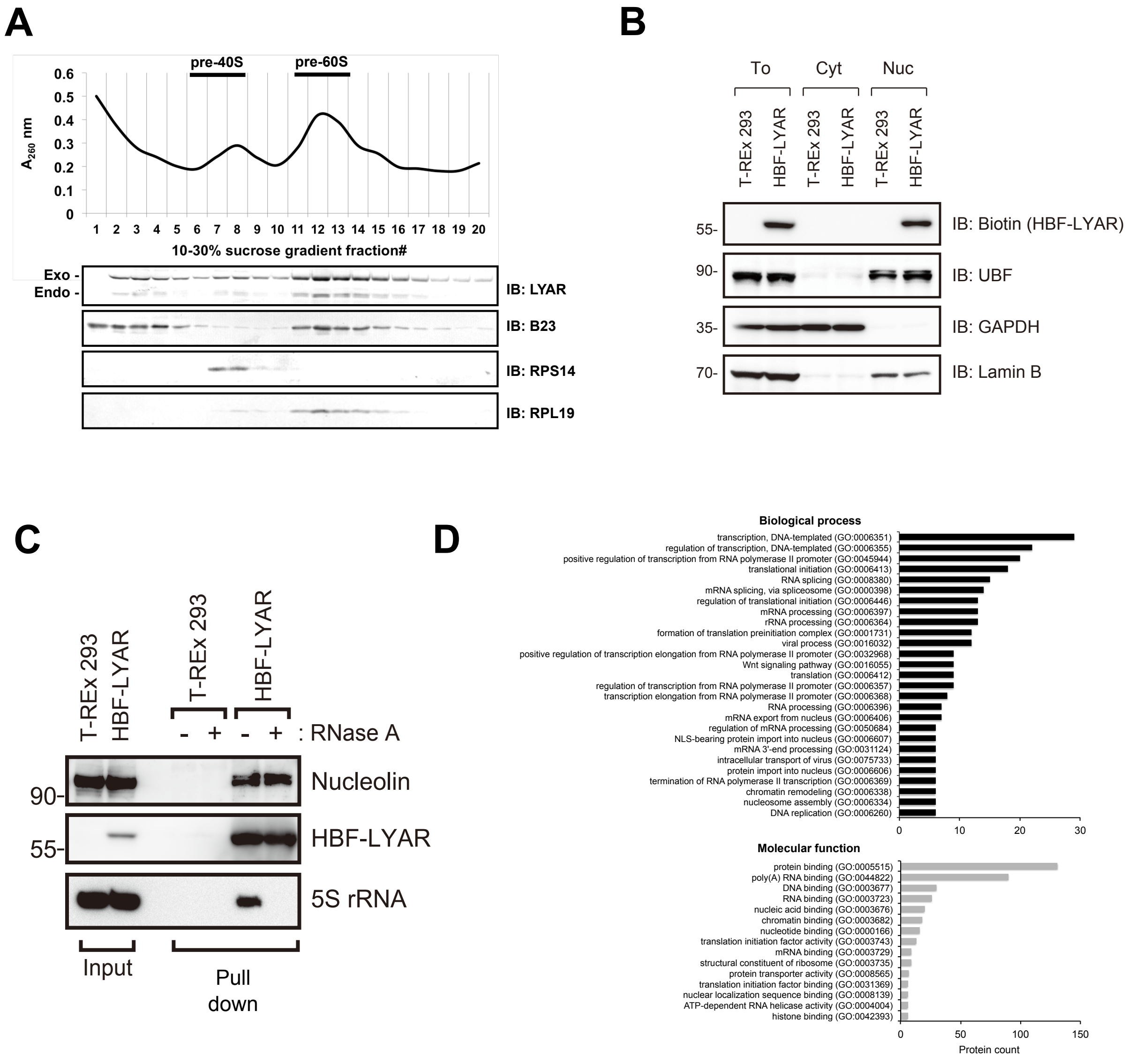

Supplementary Figure S4

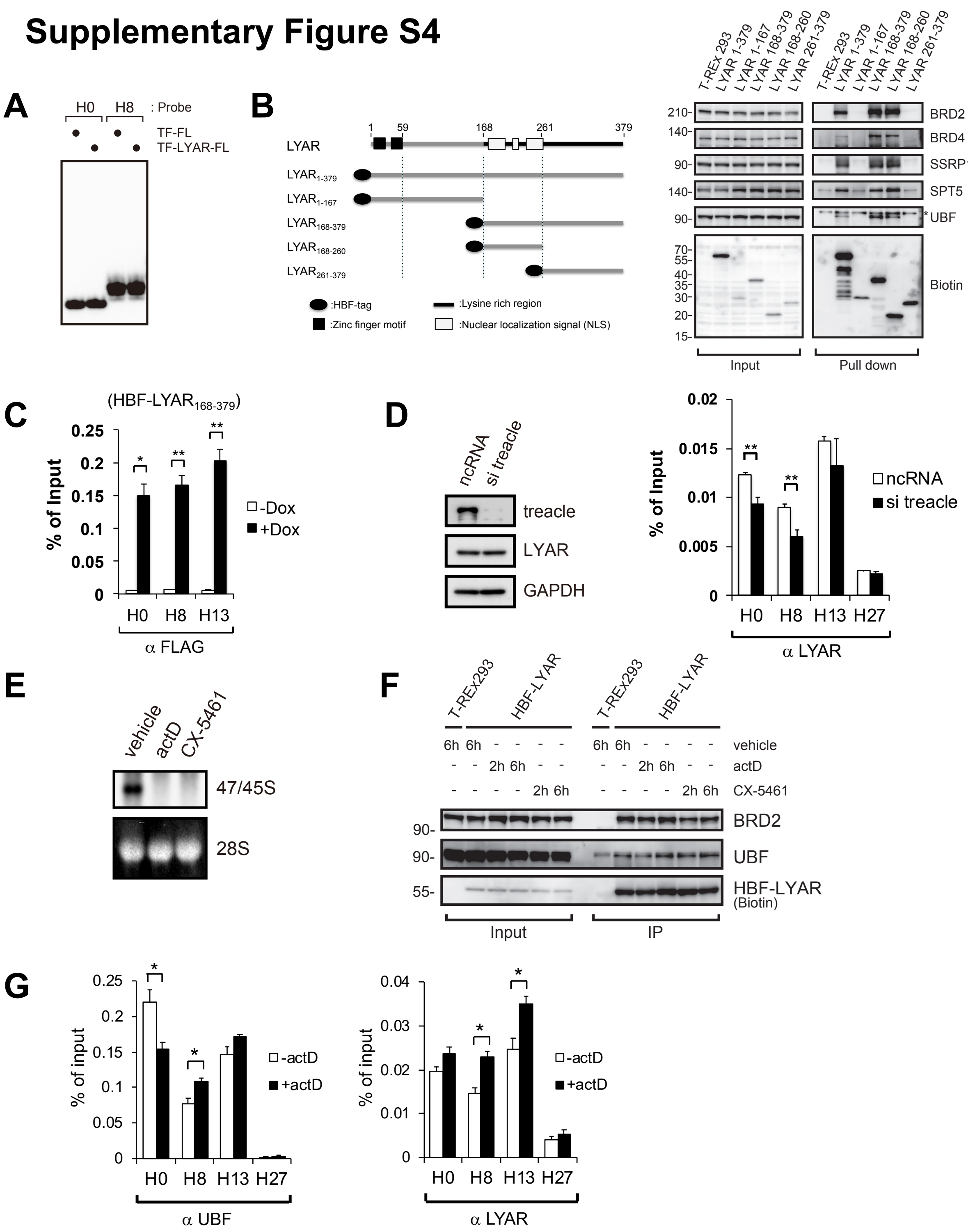

Supplementary Figure S5

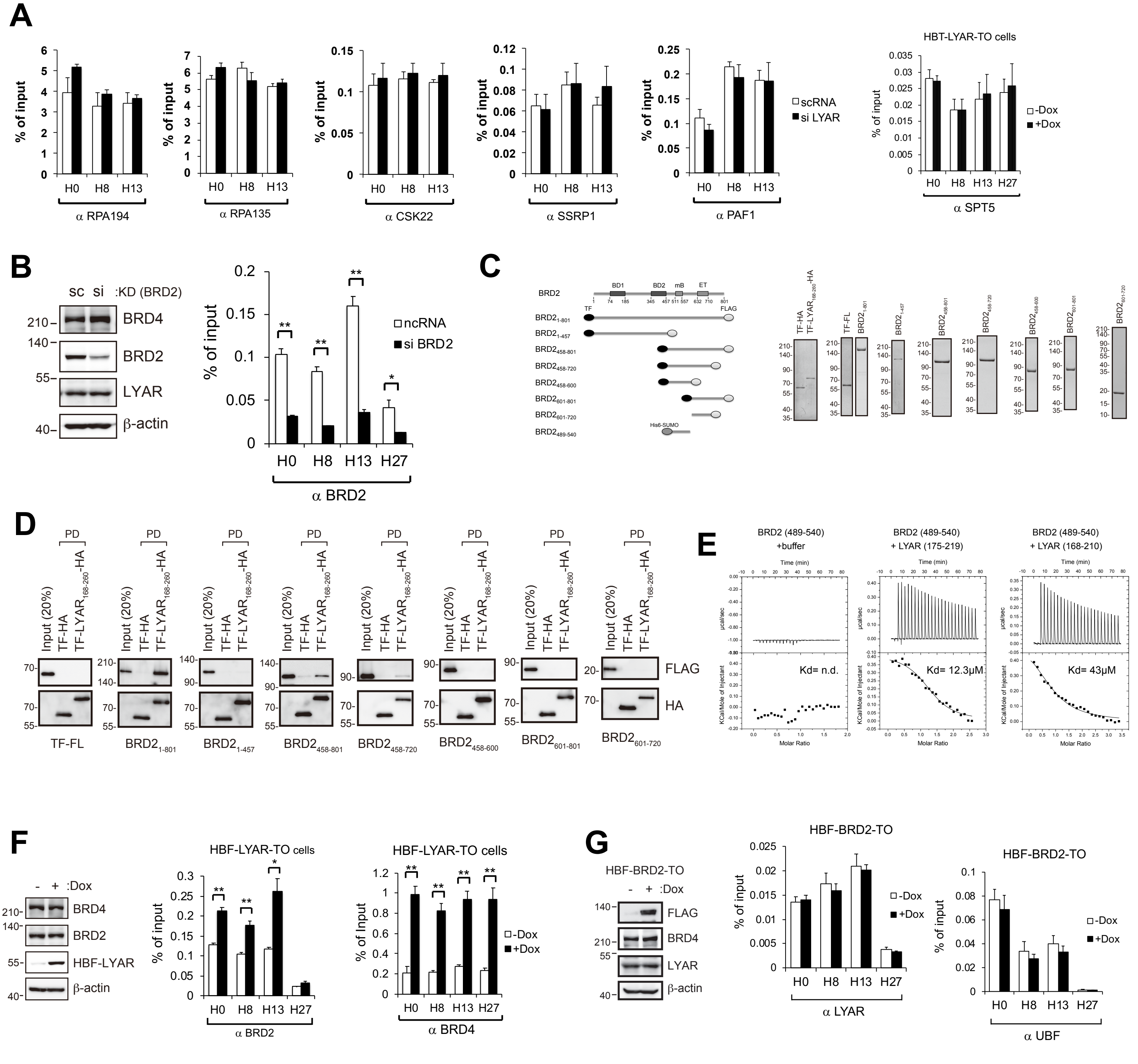

Supplementary Figure S6

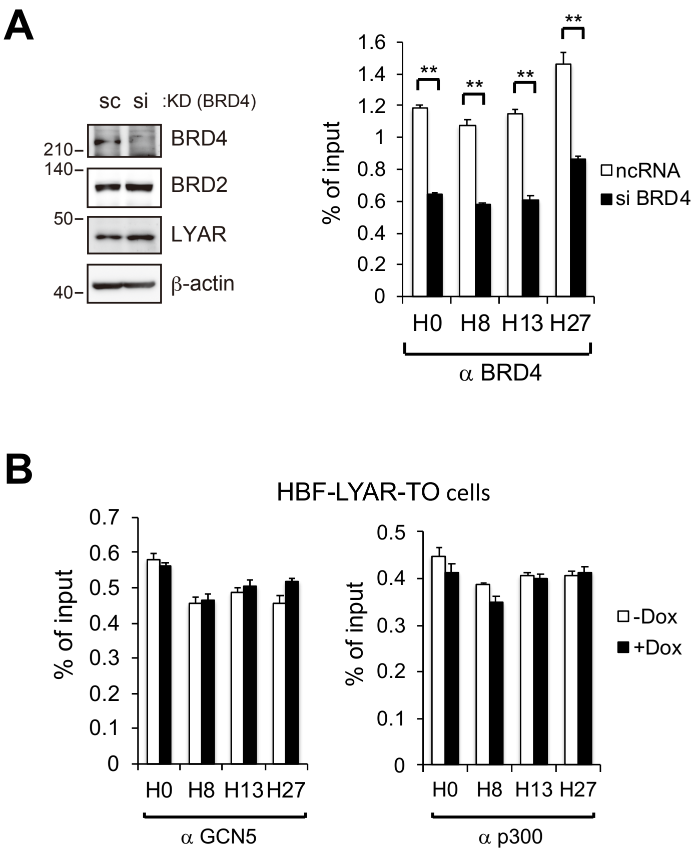

Supplement: gkz747_Supplemental_Files [file gkz747_supplemental_files.zip › Izumikawa et al Suppl Materials.pdf]
